# Human umbilical cord blood plasma as an alternative to animal sera for mesenchymal stromal cells *in vitro* expansion – A multicomponent metabolomic analysis

**DOI:** 10.1371/journal.pone.0203936

**Published:** 2018-10-10

**Authors:** A. R. Caseiro, G. Ivanova, S. S. Pedrosa, M. V. Branquinho, P. Georgieva, P. P. Barbosa, J. D. Santos, R. Magalhães, P. Teixeira, T. Pereira, A. C. Maurício

**Affiliations:** 1 Departamento de Clínicas Veterinárias, Instituto de Ciências Biomédicas de Abel Salazar (ICBAS), Universidade do Porto (UP), Rua de Jorge Viterbo Ferreira, n° 228, Porto, Portugal; 2 Centro de Estudos de Ciência Animal (CECA), Instituto de Ciências, Tecnologias e Agroambiente da Universidade do Porto (ICETA), Rua D. Manuel II, Apartado 55142, Porto, Portugal; 3 REQUIMTE/LAQV–U. Porto–Porto/Portugal, Departamento de Engenharia Metalúrgica e Materiais, Faculdade de Engenharia, Universidade do Porto, Rua Dr. Roberto Frias, s/n, Porto, Portugal; 4 REQUIMTE- LAQV, Departamento de Química e Bioquímica, Faculdade de Ciências, Universidade do Porto, Rua do Campo Alegre, Porto, Portugal; 5 Department of Electronics Telecommunications and Informatics, IEETA, University of Aveiro, Campus Universitário de Santiago, Aveiro, Portugal; 6 Biosckin, Molecular and Cell Therapies S.A., Laboratório Criovida, TecMaia, Rua Engenheiro Frederico Ulrich 2650, Moreira da Maia, Portugal; 7 Universidade Católica Portuguesa, CBQF—Centro de Biotecnologia e Química Fina–Laboratório Associado, Escola Superior de Biotecnologia, Rua Arquiteto Lobão Vital 172, Porto, Portugal; EFS, FRANCE

## Abstract

Mesenchymal Stromal cells (MSCs) have a potential role in cell-based therapies. Foetal bovine serum (FBS) is used to supplement the basal cell culture medium but presents several disadvantages and risks. Other alternatives have been studied, including human umbilical cord blood plasma (hUCBP), aiming at the development of xeno-free culturing protocols. A comparative characterization of multicomponent metabolic composition of hUCBP and commercial FBS based on Nuclear Magnetic Resonance (NMR) spectroscopy and multivariate statistical analysis was performed. The analysis of ^1^H-NMR spectra revealed both similarities and differences between the two proposed supplements. Similar metabolites (amino acids, glucose, lipids and nucleotides) were found in the hUCBP and FBS NMR spectra. The results show that the major difference between the metabolic profiles of the two proposed supplements are due to the significantly higher levels of glucose and lower levels of lactate, glutamate, alanine and branched chain amino acids in hUCBP. Similar or slightly different levels of important proteinogenic amino acids, as well as of nucleotides, lipids were found in the hUCBP and FBS. In order to validate it’s suitability for cell culture, umbilical cord-MSCs (UC-MSCs) and dental pulp stem cells (DPSCs) were expanded using hUCBP. In both hMSCs, *in vitro* culture with hUCBP supplementation presented similar to improved metabolic performances when compared to FBS. The two cell types tested expressed different optimum hUCBP percentage content. For DPSCs, the optimum hUCBP content was 6% and for UC-MSCs, 4%. Cultured hMSCs displayed no changes in senescence indicators, as well as maintained characteristic surface marker’s expression. FBS substitution was associated with an increase in early apoptosis events, in a dose dependent manner, as well as to slight up- and down-regulation of targeted gene’s expression. Tri-lineage differentiation capacity was also influenced by the substitution of FBS by hUCBP.

## 1. Introduction

Mesenchymal Stem / Stromal Cells are gaining ground as a source of innovative advanced therapeutic strategies for a number of diseases, with hundreds of ongoing or recruiting trials involving application of Human Mesenchymal Stromal Cells (hMSCs) (www.clinicaltrial.gov; database accessed on January 2018). As such, the scientific community has focused on developing and optimising protocols for their isolation, expansion, and cryopreservation aiming at their effective application in state of the art therapeutic approaches to relevant clinical situations. MSCs can be isolated from many tissue sources, such as the bone marrow, peripheral blood, dental pulp, umbilical cord, and amniotic fluid [1]. Human MSCs characteristics have been defined by the Mesenchymal and Tissue Stem Cell Committee of the International Society for Cellular Therapy [2] as being plastic-adherent when maintained in standard culture conditions, expressing cluster of differentiation (CD) 105, CD73 and CD90, and lacking expression of CD45, CD34, CD14 or CD11b, CD79α or CD19 and HLA-DR surface molecules. Finally, the hMSCs are able to differentiate into osteoblasts, adipocytes and chondroblasts *in vitro*, in the presence of adequate differentiation media [3].

The umbilical cord tissue or matrix (Wharton jelly–UCT) is an important source of MSCs, baring advantages over other sources for being non-invasively collected (previously considered as medical waste). Also, increasing numbers of high quality cryopreserved samples worldwide, in private and public cryopreservation banks, and the ready availability for therapeutic usage [4–12] adds a further advantage. Dental derived stem cells are other population with increasing relevance, mostly due to the increasing availability of cryopreservation options, for autologous and allogeneic applications, in both infants and adult individuals.

According to the European Medicines Agency (EMA) and European Commission regulation [EC 1394/2007 of the European Commission], hMSCs are considered as medicinal products and must be produced in compliance with good manufacturing practices (GMP) [13]. For that purpose, stem cells from either source must be cultured on large scale, and isolated, processed and cryopreserved according to GMP conditions. This implies the usage of xeno-free medium and reagents of clinical grade for the cell culture, for quality assurance and control, and long term rastreability of the samples [8, 14, 15].

Currently available hMSCs expansion protocols use foetal bovine serum (FBS) and other animal sera for culture media supplementation, providing vital nutrients and growth factors. These also act as a pH buffering agent, and provide protection against cytotoxic components, playing a crucial role in hMSCs survival. Nonetheless, there are several concerns on the use of FBS for clinical applications, due to the potential immunogenicity of FBS cultured cells and of xenogeneic proteins present in the cell culture, capable of inducing anaphylactic or *arthus*-like immune reactions, which have been reported. Notwithstanding this, FBS-cultured hMSCs have been approved by the US Food and Drug Administration (FDA) for medical applications [16, 17]. Complications of using animal sera for *in vitro* cell culture also include high batch-to-batch variability, unexpected cell growth characteristics, cytotoxicity of uncharacterized factors in the serum, and the risk of possible contamination with virus, prions, bacteria, nanobacteria, mycoplasma, yeast, *fungi*, and endotoxins [1, 7, 8, 18–21]. The use of animal sera also implies the sacrifice of a huge number of young animals for blood collection, entailing severe economic, ethical and scientific issues on animal welfare [8].

These issues reinforce the intensive search for new alternatives to animal sera to better comply with GMP and FDA regulations (EMEA CPMP/BWP/1793/02; 2003; EMEA/410/01 rev. 2; 2004) [22, 23]. The substituent cell culture medium supplement must match the animal sera characteristics crucial for *ex vivo* cell culture, while maintaining a fair cost, being suitable for safety and quality control, and readily/easily available. A variety of human blood products have been already considered as animal sera substituents [15, 24–26]. Amongst them, human umbilical cord blood serum or plasma (hUCBS or hUCBP, respectively), due to its rich soluble growth factors’ content. These soluble factors support the growth, proliferation, and differentiation of hMSCs and HSCs [15, 26, 27], with potential pre-clinical and clinical applications [6–8, 14, 28]. The implementation of hUCBS or hUCBP could solve important economic, ethical and scientific issues associated with FBS supplementation in cell-based therapies development. Moreover, the hUCBS and hUCBP are attractive alternatives to FBS due to the worldwide increase number of cryopreserved UCB units in Public and Private Cord Blood Banks, following high quality standards imposed by the international and national authorities [4–9, 29].

The successful employment of hUCBP can result in the preparation of fully autologous cellular therapies, for patients with cryopreserved serum/plasma and cells or tissue. Alternatively, once the regulatory requisites are established, it can be envisioned for the preparation of allogeneic cellular therapies. This would bring increased benefits for a larger number of patients, and turning cellular therapies promptly available, allowing medical action in optimal ‘windows of therapeutic opportunity’ for numerous diseases. Additionally, it would contribute to the broadening of possible beneficiaries for such therapies, including people that might have not had the chance to preserve autologous samples, and that may be in need of these advanced therapeutic strategies (for example, suffering from age-related of degenerative diseases).

In a previous study, we have identified specific components and factors (proliferative and anti-apoptotic growth factors, immunomodulatory and immunosuppressive cytokines and chemokines) in hUCBP that could have promoting effects on hMSCs *in vitro* expansion. The results strongly suggest that hUCBP might be an alternative for the FBS supplemented culture medium used in hMSCs *in vitro* isolation, expansion and cryopreservation [4, 8]. This assumption converges with the recently published results on the effect of haemoderived supplements on the hMSCs growth rate, viability and differentiation properties [15, 26, 30–33]. For this purpose, a detailed analysis of the hUCBP components is crucial to validate this human haemoderivate by-product as a supplement for *ex vivo* expansion and processing of hMSCs for clinical applications.

Metabolomics is an important and well-established analytical approach for the comprehensive and systematic determination of metabolite levels in biological samples. Nuclear magnetic resonance (NMR) spectroscopy is one of the main tools for metabolic profiling, identification and quantification of known and unknown metabolites in bio-fluids, tissues and cells. [34–36]. All metabolomics studies including NMR-metabolomics, result in a large number of complex multivariate data sets that require chemometric and bio-informatics methods for accurate data interpretation, visualization and analysis [37, 38]. Data-reduction and pattern recognition techniques such as principal component analysis (PCA) allow for analysis, interpretation and classification of the large number of complex NMR spectroscopic data usually acquired for metabolic profiling of biological samples [34–36, 39, 40].

The first part of this study aims at the characterization of the metabolite composition of hUCBP, to address its capacity as a culture medium supplement and FBS substituent to support xeno-free *in vitro* proliferation of hMSCs. The second part of the study aimed at the validation of hUCBP substitution for the expansion of umbilical cord (UC-MSCs) and dental pulp- derived mesenchymal stem cells (DPSCs), and its effects on cell viability and senescence, surface marker- and gene expression, as well as on their tri-lineage differentiation capacity.

### 2. Material and methods

The hUCB samples included in the present study were processed and cryopreserved by Biosckin, Molecular and Cell Therapies S.A. (Maia, Portugal), which is an authorized Portuguese Private Cord Blood Bank for UCB processing and cryopreservation by the Direção-Geral de Saúde (DGS), and certified for ISO9001 and for NP4457 (Authorization from DGS in attachment). The hUCB was collected from healthy donors that signed a contract with Biosckin, Molecular and Cell Therapies S.A. for processing and cryopreserved the buffy coat of the collected umbilical cord blood where the hematopoietic stem cells are included. On the other hand, the hUCB plasma is not cryopreserved and it is a by-product which is not cryopreserved and it is usually destroyed. The present experimental work used those hUCB plasmas and it should be emphasized that the donors that have this contract with Biosckin, Molecular and Cell Therapies S.A. authorize the use of these by-products for research. Patient records used in our retrospective study were fully anonymized before the authors accessed the data for the present study and the ethics committee waived the requirement for informed consent. Also, there is a scientific protocol / contract between the University of Porto and the Biosckin, Molecular and Cell Therapies S.A., which includes I&D projects, PhD and master supervision of students, among other training and teaching activities. For that reason, it was not necessary to submit this study to approval of the ethical commission.

### 2.1. Study design

In the first part of the study, appropriate 1D and 2D NMR techniques have been used to identify metabolites in the hUCBP (a total number of 13 samples collected from healthy donors) and two commercial FBS products (FBS_I and FBS_II), and to define their relative quantitative distribution. Principal component analysis has been used to interpret and classify the NMR spectral data and to evaluate the main factors contributing to the discrimination between these two groups of samples. Prior to NMR analysis, a complete characterization of the hUCB samples was performed (haematology for total nucleated cells (TNC) and white blood cells (WBC) count, flow cytometry for CD45^+^ / CD34^+^ cell counts, and cell viability), accompanied by pre-birth and immediate after-birth maternal screening for relevant transmittable pathogens, including Cytomegalovirus, Venereal disease/ Syphilis, Hepatitis B and C, HIV-I and II, Toxoplasmosis (and Human T-Cell Lymphotropic Virus Types I and II (HTLV-I/-II), if relevant). Microbiological analysis was performed to hUCB units after volume reduction using an automated blood culture system for aerobic and anaerobic microorganisms and *fungi*.

In the second part of the study, the capacity of hUCBP to sustain both hMSCs expansion was assessed with PrestoBlue^®^ viability assay at different time points [24h, 72h, 120h, 168h, 216h], using umbilical cord-MSCs (UC-MSCs) and dental pulp stem cells (DPSCs). Crescent concentrations of hUCBP were added as a supplement of the basal culture medium [2%, 4%, 6%, 8%], and compared with FBS_II at 10%, generally used standard culturing protocols. The effects of the supplement substitution on hMSCs cultures were assessed regarding senescence and apoptosis events, hMSCs’ phenotypical identity/ surface marker and gene expression, as well as tri-lineage differentiation capacity.

### 2.2. Ethics and regulation

The hUCB included in the present study were processed and cryopreserved by Biosckin, Molecular and cell Therapies S.A. (Maia, Portugal), an authorized Portuguese Private Cord Blood Bank for UCB processing and cryopreservation by the Direcção-Geral de Saúde (DGS), and certified for ISO9001 and for NP4457. The hUCB was collected from healthy donors, according to Netcord guidelines and following Portuguese laws N° 12/2009 and N° 1/2015 (Diário da República, lei 12/2009 de 26 de Março de 2009 and Diário da República, lei 1/2015 de 8 de Janeiro de 2015), with written informed consents according to Directive 2004/23/EC, which sets the standards of quality and safety for the donation, procurement, testing, processing, preservation, storage and distribution of human tissues and cells.

### 2.3. Preparation of umbilical cord plasma

Thirteen samples of hUCBP collected from different healthy donors were used for ^1^H-NMR analysis (N = 13). The hUCBP samples were analysed by haematology auto-analyser, flow cytometry and for microbiological contamination for aerobic and anaerobic microorganisms and *fungi*. A pool from 12 out of 13 of these hUCBP (N = 12) was used for the *in vitro* validation with UC-MSCs and DPSCs viability assessment.

### 2.4. Donors selection and umbilical cord blood (UCB) collection

Maternal and neonatal pairs were evaluated during the antenatal period in the maternity wards at different hospitals collaborating with the Umbilical Cord Blood Private Bank of Biosckin, Molecular and Cell Therapies, S.A. (Maia, Portugal). Donors signed informed consent before delivery and were clinically evaluated according to Portuguese law (Diário da República, lei N° 12/2009 de 26 de Março de 2009 and lei N° 1/2015 de 8 de Janeiro de 2015). Human UCB was collected from the umbilical vein by gravity into a 150 ml volume simple bag (reference #1385.13, Suru, Portugal) containing 21 ml of citrate-phosphate-dextrose (CPD). The hUCB was stored at 4°C ± 2°C until processing for cryopreservation. Human UCB samples were transported to the Biosckin, Molecular and Cell Therapies S.A. laboratory at refrigerated temperatures ranging between 4°C and 22°C, within 72 hours of collection. The collection method was the same for all the units included in this study.

### 2.5. Volume reduction with the AXP automated system

The AXP system^®^ (Thermogenesis) was used to achieve the separation of a concentrated mononuclear cell (MNC) fraction of uniform volume. During the two-step centrifugation, whole blood is separated into three layers that are delivered into a red blood cells (RBC) bag and a freezing bag. Plasma remains in the processing bag which is also the plasma bag. The programmed final volume in the cryopreservation bag was 21 ml. Samples of the hUCB were taken by sampling pillows integrated within the kit for hematology auto-analyzer and flow cytometry analysis [8, 41–43].

### 2.6. Haematology auto-analyzer, flow cytometry and microbiological analysis of the UCB

Maternal screening for Cytomegalovirus, Venereal disease/ Syphilis, Hepatitis B and C, HIV-I and II, Toxoplasmosis (and Human T-Cell Lymphotropic Virus Types I and II (HTLV-I/-II), if relevant) was mandatory for sample acceptance for processing. All cryopreserved samples were negative to the screened agents. Microbiological analysis was performed after volume reduction and before cryopreservation (samples are maintained in quarantine until final results are disclosed) using an automated blood culture system (BacT/ALERT^®^, BioMérieux) at 35°C for 14 days, and included microbiologic analysis for aerobic and anaerobic microorganisms and *fungi*. For the UCB units that presented microbial contamination, the microorganism was identified and was performed the antibiogram for identification of the antibiotic sensitivity of the identified microorganisms [8].

Total nucleated cells (TNC) and the number of white blood cells (WBC) were counted with a hematology auto-analyzer (Ac T diff2™, Beckman Coulter, Inc.) before and after the AXP volume reduction procedure. These counts were not corrected for nucleated RBC. The CD34^+^ cell number and viability were quantified by flow cytometry (BD FACSCalibur™ 3 CA Becton Dickinson, BD Biosciences), the software for acquisition and analysis were BD CellQuest™ and BDCellQuest Pro Templates, respectively. The clusters of differentiation used to enumerate the total number of CD34^+^ cells and the total number of leucocytes (CD45^+^), the 7-Amino-Actinomycin D (7AAD) nucleic acid dye was used for viability measure (BD Stem Cell Enumeration kit, Becton Dickinson, BD Biosciences), according to the manufacturer’s protocol. The BD Stem Cell Enumeration simultaneously enumerates the total viable dual-positive (CD45^+^/CD34^+^) hematopoietic stem cells in absolute counts of CD34^+^ cells per μL and the percentage (%) of viable leucocytes (CD45^+^) that are CD34^+^ [8].

### 2.7. FBS commercial samples

The two FBS formulations commercially available as a supplement for hMSCs culture routinely used by our research group for *in vitro* isolation and expansion were tested. The FBS_I was obtained from GIBCO (Gibco^®^ Invitrogen, EU Approved FBS, Origin South America, Heat Inactivated, and reference #10500–064). The FBS_II was obtained from BI, Biological Industries (BI LTD, Certified FBS, reference #04-400-1A), and is classified for human MSCs, 0.1 μm membrane filtered, tested for mycoplasma, and virus screened. This FBS_II is heat inactivated, sterile-filtered, and according to the manufacturer information, presents hemoglobin in a concentration ≤25 mg/dl, and ≤10 EU/ml endotoxin, and it was used for the *in vitro* validation. For both formulations, 3 different samples obtained from a different lot were tested in the ^1^H-NMR experiments (N = 3).

### 2.8. Part I: Supplements metabolomic characterization

#### 2.8.1. NMR spectroscopy

A 600 μl aliquot of each sample was transferred into 5 mm NMR tubes and mixed with 50 μl deuterium oxide (D_2_O) containing 0.05mM sodium trimethylsilyl-[2,2,3,3-d4]-propionate (TSP) as an internal reference. The NMR experiments were recorded at 300K on a Bruker Avance III 600 HD spectrometer, equipped with CryoProbe Prodigy. All ^1^H NMR spectra were acquired with water suppression using a 1D NOESY (noesygppr1d) and a Carr-Purcell-Meiboom-Gill (CPMG, cpmgpr1d) pulse sequences [44, 45]. The 1D NOESY spectra were collected using 5s relaxation delay and mixing time of 0.01s. The CPMG experiments (cpmgpr1d) were acquired with relaxation delay 4s, during which the water resonance signal was selectively irradiated. The echo time was optimized for each sample and was between 0.3 and 0.8 ms, and a loop for T2 filter of 20 was used. For all ^1^H-NMR spectra 128 or 256 transients of a spectral width of 10000 Hz were collected into 32 K time domain points. The spectra were processed with the Bruker Topspin software package (version 3.2). The time domain data were multiplied by an exponential function with line-broadening factor of 0.3 Hz and zero-filled to 64 k prior to Fourier transformation. Two dimensional (2D) ^1^H/^1^H COSY and TOCSY spectra were recorded in phase sensitive mode and with water suppression; a relaxation delay of 2s, 16 or 32 scans, a total 2K data points in F2 and 256 or 512 data points in F1 over a spectral width of 10000 Hz. 2D ^1^H/^13^C heteronuclear single quantum coherence (HSQC) experiments were carried out with a spectral width of 10000 Hz for ^1^H and 27000 Hz for ^13^C, relaxation delay 1.5 s, Fourier transform (FT) size 2Kx1K. The quantitative distribution of NMR-detectible metabolites in hUCBP and FBS (FBS_I and FBS_II) analysed samples was determined from the integral intensity of characteristic signals in ^1^H-NMR spectra of the samples referenced to the total integral intensity of the signals in the spectra, considering the number of the contributing nuclei for that particular resonance signal.

#### 2.8.2. Multivariate statistical analysis of NMR spectroscopy data

In the present study, the integral intensities over small chemical shift regions (bins) of size 0.01 ppm over the spectral region between 0.50 and 9.00 ppm were used as descriptors for interpretation and statistical analysis of the NMR data. Data was normalized by the total sum of the integral intensities in the spectral region of interest. In all studies the water and citric acid spectral regions were excluded. Additionally, a separate NMR-data set was prepared with the regions containing sugars and lactate resonance signals excluded. The Principal Component Analysis (PCA), an unsupervised pattern recognition approach, was applied to examine the intrinsic variation in the data set and compare the metabolic profiles of the 13 hUCBP samples and 2 commercial formulations of FBS (FBS_I and FBS_II) [46]. PCA determines the independent sources of variance across a set of spectra and ranks these Principal Components (PCs) in order of their contribution to the overall variance. PCA facilitates the identification of the sources of variation relevant to systematic changes in a number of key features in the NMR spectra. Based on the singular values decomposition (SVD) technique, Principal Components (PCs) are extracted from the initial NMR data matrix. Each PC is orthogonal (uncorrelated) with all other PCs. The PCs are ordered based on their information content, with the first few PCs containing the largest part of the variance of the data set. Two dimensional PC maps (PC1 vs. PC2) were used as a convenient way to visualize inherent data clusters and outliers, and make conclusions on the degree of correlation between the hUCBP and both FBS samples. If there are systematic metabolic differences between samples, similar samples may cluster together in the scores plot, and classification of the samples may be possible. Further, PC loading plots (ppm vs. PC1 or PC2) were used to reveal the main metabolites and the associated spectral regions responsible for the NMR data variability. The loading plots define the contribution of variables, i.e. the metabolite content for the divergence or clustering between the samples [34–36].

### 2.9. Part II: *In vitro* validation

#### 2.9.1. Cell viability assessment

**Cell culture and maintenance:** UC-MSCs and DPSCs were thawed and *in vitro* expanded using standard protocols previously reported by our research group [4–12, 29, 47–49]. UC-MSCs and DPSCs were maintained at 37°C and 95% humidified atmosphere with 5% CO_2_ environment. UC-MSCs were obtained from PromoCell, Cat. C-12971; Lot No. 1112304.2) and DPSCs from AllCells, LLC (Cat. DP0037F, Lot No. DPSC090411-01). MSCs’ batches identity was confirmed through flow cytometry analysis. Passage 5–7 cultures were utilized in the presented assays in both cell populations. Both populations were initially maintained in αMEM, with GlutaMAX™, without nucleosides (Gibco, reference #32561029) supplemented with 10% (v/v) FBS, 100 IU/ml penicillin, 0.1 mg/ml streptomycin (Gibco, reference #15140122), 2.05 μg/ml amphotericin B (Gibco, reference #15290026) and 10 mM HEPES Buffer solution (Gibco, 15630122). For the *in vitro* viability studies, the FBS_II was selected for culture medium supplementation of both hMSCs types under study.

**PrestoBlue Cell Viability Protocol:** The PrestoBlue (Thermo Fisher Scientific, Molecular Probes) assay is a commercially available, ready-to-use, water-soluble preparation. Cells were seeded in 24 well plate at 4x10^3^ cells/well. The cells were left adhering in complete culture medium, overnight. After this period, culture media was replaced by test media [αMEM with GlutaMAX, without nucleosides supplemented with either 2%, 4%, 6%, or 8% of hUCBP pool or 10% FBS (v/v), 100 IU/mL penicillin, 0.1 mg/ml streptomycin, 2.05 μg/ml amphotericin B and 10 mM HEPES buffer solution]. Previous experimental set ups revealed jellification of the hUCBP supplemented medium, despite of its collection and transportation in CPD containing collection bags. Hence, heparin (Heparin Sodium, 5.000 IU/ml, BBraun^®^) was added to the hUCBP supplemented medium formulations, at 2 UI/ml. Preliminary assays presented no significant effect of heparin addition on standard culture conditions, using 10% FBS supplemented αMEM (data not shown).

At every time point [24 hours (1 day), 72 hours (3 days), 120 hours (5 days), 168 hours (7 days), and 216 hours (9 days)], the culture media was removed and fresh adequate culture media was added to each well, with 10% (v/v) of 10x PrestoBlue cell viability reagent (Thermo Fisher Scientific, Molecular Probes, Invitrogen, reference #A13262), and UC-MSCs and DPSCs were incubated for 1 hour at 37°C, 5% CO_2_. Changes in cell viability were detected by absorbance spectroscopy of cell culture supernatant, and absorbance was read at 570nm and 595 nm in a Thermo Scientific Multiskan FC plate reader. Absorbance readings were normalized and data corrected to unseeded control wells’ readings. At each time point, sample wells were harvested using Trypsin-EDTA and triplicate cell counts were performed with Trypan Blue exclusion dye assay (15250061, Gibco), using an automated counter (Countess^TM^ II FL Automated Cell Counter, Invitrogen).

#### 2.9.2. Senescence and apoptosis

To assess for signs of senescence in the cellular populations, β-Galactosidase activity was assayed. Cells were plated in 96-well plates, as described for the growth dynamics assessment, maintaining the assigned hUCBP or FBS supplemented media. At 3, 5 and 7 days [72, 120 and 168 hours], culture media was removed, and cells were washed once with DPBS and incubated with β-Galactosidase Assay Reagent (75705, Thermo Fisher Scientific), for 30 minutes at 37°C. Absorbance was read at 405 nm. UC-MSCs and DPSCs cultured in either hUCBP or FBS supplemented media were further assessed for apoptosis events, through the detection of Annexin V and Propidium Iodine (PI) staining (BMS500FI, eBioscience). For such, cells cultured for 5 days in each condition were harvested using Trypsin-EDTA, counted and resuspended in provided Binding Buffer. Cells were incubated with Annexin V-FITC and PI, as per manufacturer’s protocol, and FACS analysis was performed using a Coulter Epics XL Flow Cytometer (Beckman Coulter Inc., Miami, FL, USA). Flow cytometry data was processed using FlowJo Engine X10.4 (v3.05478, LLC).

#### 2.9.3. MSCs’ Phenotype identity

The surface marker profiles of UC-MSCs and DPSCs cultured in either hUCBP or FBS supplemented media were assessed though Flow Cytometry. MSCs populations were cultured for 5 days, as described previously and harvested using Accutase^TM^ Cell detachment solution (561527, BD Biosciences), counted and resuspended in Stain Buffer (554676, BD Biosciences). Cells were incubated with anti-positive (CD90, CD105, CD44) and negative marker (CD34, CD11b, CD19, CD45, MHC class II) antibodies and assayed as per manufacturer’s instructions (Human MSC Analysis Kit, 562245, BD Biosciences), using a BD FACSCalibur 3 CA Becton Dickinson, BD Biosciences. Flow cytometry data was processed using FlowJo Engine X10.4 (v3.05478, LLC).

#### 2.9.4. Gene expression

Reverse transcriptase Polymerase chain reaction (RT-PCR) and qPCR was performed targeting housekeeping and MSCs’ related gene sequences listed in **Table 1**.

**Table 1:** Primer sequence information for qRT-PCR custom plate array.

*Gene*	*Length (bp)*	*Bio Rad Assay ID*
*MSCs targets*		
***CD34***	99	qHsaCID0007456
***CD90/ THY1***	120	qHsaCED0036661
***CD73/ NT5E***	117	qHsaCID0036556
***CD105/ ENG***	124	qHsaCID0010800
***CD166/ ALCAM***	173	qHsaCID0037887
***CD117/ c-kit***	63	qHsaCID0008692
***SOX2***	98	qHsaCED0036871
***OCT3-4/ POU5F1***	100	qHsaCED0038334
***MHC class I/ HLA-A***	115	qHsaCED0037388
***MHC class II/ HLA-DRA***	119	qHsaCED0037296
*Housekeeping genes*:		
***β-actin***	62	qHsaCED0036269
***GAPDH***	117	qHsaCED0038674

MSCs populations cultured in the described media were harvested using Trypsin-EDTA, as previously described, and pellets of 10^6^ cells of each group were used for total RNA extraction, using the Aurum^TM^ Total RNA Mini kit (732–6820, Bio Rad). Briefly, cell pellets were lysed, DNA was removed with DNase I enzyme and obtained RNA was eluted. Total RNA was quantified using a nanophotometer readings at 260 and 280 nm (NanoPhotometer^TM^ Pearl, Implen GmbH). Once RNA concentrations were tuned between samples, cDNA was synthesized from the purified RNA using the iScript Reverse Transcription kit (170–8891, Bio Rad) and T100 Terma Cyclar Thermocycler (Bio Rad), as per manufacturer’s instructions. Quantitative PCR (qPCR) was performed in a CFX96 Touch^TM^ (BioRad) apparatus using the iTAQ^TM^ SYBR® Green Supermix (172–5120, BioRad) and custom PCR plates encompassing duplicates of targeted human genes and negative control (**Table 1**). Recommended PrimePCR cycling protocol was employed: 95°C for 2 minutes (activation), 40 cycles comprising 95°C for 5 seconds (denaturation) -60°C for 30 seconds (annealing), and 65 to 95°C (0.5°C increments), 5 secs/step (melt curve). The number of cycle threshold for each well was recorded. Data was processed using BioRad CFX^®^ Manager Software 3.1 (Bio Rad Laboratories). Fold differences were calculated using the standard ΔΔCq method with GAPDH and β-actin as housekeeping genes. The reference gene on the hUCBP supplemented samples was calculated relative to calibrator sample (FBS10%) (fold change between two Cq values) through the equation: Relative quantification (RQ) = 2^ΔΔCt^

#### 2.9.5 Multilineage differentiation

**Osteogenic and Adipogenic differentiation protocols**—MSCs were seeded onto 24-well plates (8.000 viable cells/cm^2^), and cultured with either FBS or hUCBP supplemented media until 70–80% confluence was reached (after 3 days). At this point, cells were transitioned to specific differentiation media: Adipogenesis (StemPro Adipogenesis Differentiation kit, A10070-01, Gibco), following manufacturer’s instructions, or formulated Osteogenesis medium, composed of expansion media further supplemented with 5nM Dexamethasone (D8893 –Sigma Aldrich^®^), 250 μM de ascorbic acid-2- phosphate (AsA2-P, A4403, Sigma Aldrich)) e 10mM β-glycerphoshate (β-GP, G9422, Sigma Aldrich). Xeno-free Osteogenic formulations were prepared using 4%, 6% or 8% hUCBP supplementation [50]. Control wells were maintained in culture media absent osteogenic supplements. Media were changed every 3 days, for 21 days, for Adipogenesis and Osteogenesis, respectively. After 14 days, cells under Adipogenic differentiation were stained with Oil Red O, for lipid droplets detection and semi-quantification. Alizarin Red S Assay was employed to asses for Osteogenic differentiation after 21 days; **Oil Red O (Qualitative and Semi-Quantitative):** Cells were fixated with 4% formaldehyde (3.7–4% buffered to pH7, 252931.1315, Panreac AppliChem) and incubated with Oil Red O (O0625, Sigma-Aldrich) working solution for 10–20 minutes at room temperature. Oil Red O was discarded and excess dye removed. Differentiated cells were observed under inverted microscope (Axiovert 40 CFL, Zeiss). For semi-quantification, Oil Red O stain was eluted through the addition of 100% isopropanol (59300, Merck Milipore^®^), and absorbance was read at 570 nm; **Alizarin Red S (Qualitative and Semi-Quantitative):** ARS Assay was used to qualitatively and semi-quantitatively determine Osteogenic differentiation of hMSCs, as described in [50]. Cells were fixed with 4% formaldehyde and stained with 40 mM ARS (2003999, Milipore) and incubated for 30 min with gentle shaking. Following incubation, unbound dye was removed and wells were washed with diH_2_O until supernatant became clear. Red stained mineralized layers produced by differentiated cells were observed macroscopically and under inverted microscope. For the extraction and quantification of the ARS, 10% acetic (ARK2183, Sigma-Aldrich) was added to the wells. Cells and mineral deposits were scraped from the plate and collected, heated at 85°C for 10 minutes and immediately transferred to ice, for 5 minutes. The samples were centrifuged, and individual absorbance values were measured at 405 nm, along with a standard curve for the calculation ARS concentration (μM).

**Chondrogenic differentiation protocol:** MSCs were suspended at 2x10^4^ viable cells/well of 96-well plate, and let to adhere for 48 hours. Chondrogenic culture medium (StemPro Chondrogenesis Differentiation kit, A10071-01, Gibco) was added to each well. Control wells remained in FBS or hUCBP supplemented media. Media was replaced every other day in both groups. After 14 days, wells were stained with Alcian Blue to assess for Proteoglycan’s synthesis by differentiated chondrocytes and Sulfated Glycosaminoglycans (GAGs) production was quantified using the Blyscan™ Glycosaminoglycan Assay (Biocolor, UK). **Alcian Blue Staining (Qualitative):** Cells were fixated with 4% formaldehyde, and stained with Alcian Blue (A9186, Sigma) solution. Alcian Blue was discarded and wells were rinsed with acetic acid 3% (v/v). Acidity was neutralized with distilled water, and left in each well for visualization. Differentiated cells were observed under inverted; ***GAGs Production (Quantitative)*:** Sulfated GAGs Assay was carried as per manufacturers’ instructions. Culture media was removed from each well and reserved. GAGs in the ECM produced by cultured cells was extracted using Papain (P3125 Sigma-Aldrich) and added to the previously collected supernatant, for total GAGs quantification). After centrifugation and removal of cellular debris, Blyscan dye reagent was added and incubated for 30 minutes, and then centrifuged to isolate the precipitated coloured complex. The precipitate was dissolved and individual absorbance values were measured at 656 nm, along with a standard curve for the extrapolation of GAGs concentration (μg/ml).

### 2.10. Statistical analysis

Statistical analysis was performed using the GraphPad Prism version 6.00 for Mac OS X (GraphPad Software, La Jolla California USA). The experiments were performed in triplicates and the results were presented as Mean ± SEM. Significant differences are also presented as percentage difference, in which 100% viability corresponds to the non-treated cells. Dunnett's multiple comparisons test against control group (10% FBS) were performed by one-way ANOVA supplemented with Tukey’s HSD post-hoc test. Quantitative data of β-Galactosidase activity, Flow cytometry data and Multilineage differentiation assays were subjected to one-way ANOVA test, followed by pairwise comparisons using Tukey’s HSD post hoc test. Statistical significance was established as P<0.05. Differences were considered statistically significant at P < 0.05. Significance of the results is indicated according to P values with one, two, three or four symbols (*) corresponding to 0.01≤P<0.05; 0.001≤P< 0.01; 0.0001≤P<0.001 and P<0.0001, respectively.

## 3. Results

Results obtained are herein presented, and the most relevant findings highlighted.

### 3.1. Haematology auto-analyser, flow cytometry and microbiological analysis of the umbilical cord blood (UCB) units

**S1 Table** (found in Supporting information) summarizes the data obtained by haematology auto-analyzer, which includes the initial WBC count, performed before the volume reduction with the AXP automated system (in cells x 10^9^ per L) and the final WBC count (in cells x 10^9^ per l), performed after the volume reduction procedure of the UCB samples. The obtained mean values were 9.61 ± 2.96 x 10^9^ cells/l and 29.42 ± 14.79 x 10^9^ cells / l, respectively. The samples presented an average of 66.17 ± 60.19 viable CD34^+^ cells/μl, with 93.13 ± 6.65% viability, and 17.63 ± 7.27 x 10^9^ viable CD45^+^/l, with 89.72 ± 4.96% viability (N = 13), demonstrating individual variability considering the UCB hematopoietic stem cells concentration and CD45^+^ / CD34^+^ cell viability. This individual variability has been previously reported in UCB units processed by AXP automated system [41–43].

Considering the microbiological evaluation of the UCBP, all the samples were negative for aerobic, anaerobic microorganisms and *fungi*, except for the sample hUCBP#5 which was positive for *Actinomyces meyeri* in anaerobiosis. Although contamination was identified in this samples, all of the haematological parameters remained within the average counts (**S1 Table,** hUCBP#5 sample). The bacterial contamination did not interfere with ^1^H-NMR analysis and no statistical differences were observed between plasma sample hUCBP#5 and the mean values calculated for the 13 plasma samples analysed (P<0.05) **(S1 Table)**. However, hUCBP#5 was excluded from the pool used for the *in vitro* validation, in order to prevent the cell culture contamination, and the hUCBP pool included only 12 out of 13 samples analysed (N = 12).

### 3.2. Part I: Supplements metabolomic characterization

#### 3.2.1. NMR spectroscopy

**Fig 1:**
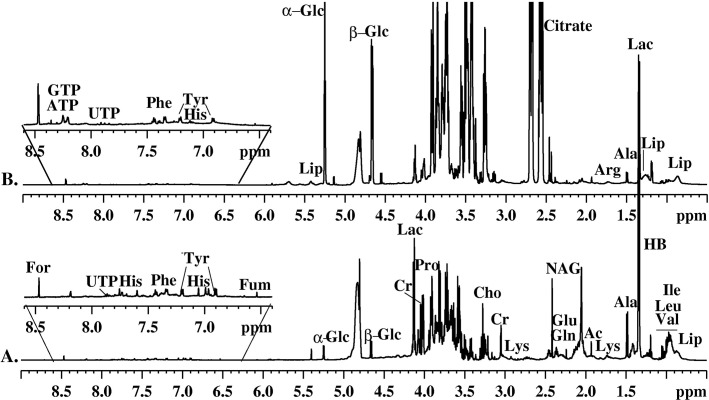
MHz ^1^H-NMR spectra of FBS_I and FBS_II and hUCBP. Average 600 MHz ^1^H-NMR spectra (A) of FBS_I, and FBS_II and (B) hUCBP samples.

^1^H-NMR spectra of all hUCBP and FBS samples were acquired with water suppression using 1D NOESY and CPMG pulse sequences. The implementation of 1D NOESY has led to ^1^H-NMR spectra with improved solvent peak (at 4.70 ppm) suppression but complex line shapes and highly overlapped resonances from the low molecular weight metabolites and macromolecular components. To reduce the complexity of the spectra, T2-edited CPMG experiments with appropriate T2 delays were used to attenuate the broad signals from high molecular weight species and facilitate the identification of small molecular components. The average ^1^H (CPMG) NMR spectra of hUCBP and FBS samples are shown in **Fig 1A** and **Fig 1B**, respectively. The assignment of the resonance signals in the spectra was based on results obtained from various 1D and 2D (^1^H/^1^H COSY, ^1^H/^1^H TOCSY, ^1^H/^13^C HSQC) NMR experiments and reference data [51, 52]. Characteristic resonance signals of metabolites identified in ^1^H -NMR spectra of hUCBP and FBS samples are labelled in **Fig 1**. The analysis of ^1^H-NMR spectra has allowed the identification and quantification of a number of metabolites in hUCBP and FBS listed in **Table 2**.

**Table 2:** Relative quantitative distribution of metabolites observed in the ^1^H-NMR spectra of hUCBP, FBS_I, and FBS_II samples.

*Metabolites*	*Code*	*Group*	*Chem*. *Shifts (ppm)*	*hUCBP*			*FBS_I*			*FBS_II*		
***1-Methylhistidine***	1^_^Me-His	CH	7,80–7.86	0,47	±	0,23	0,43	±	0,05	0,34	±	0,08
***3-Methylhistidine***	3-Me-His	CH	7,60–7,68	0,47	±	0,21	0,43	±	0,06	0,31	±	0,09
***Acetate***	Ace	CH_3_	1,93	0,14	±	0,05	0,18	±	0,01	0,18	±	0,02
***Adenosine***	ADP/ATP	CH	8,22	0,48	±	0,21	0,41	±	0,06	0,28	±	0,09
***Alanine***	Ala	CH_3_	1,46	0,35	±	0,12	0,86	±	0,04	0,89	±	0,02
***Bile Acids***	BA	CH_3_	0,50–0,75	0,37	±	0,16	0,26	±	0,02	0,21	±	0,07
***Choline***	Cho	NCH_3_	3,24	0,13	±	0,04	0,12	±	0,00	0,11	±	0,01
***Citrulline***	Citr	CH_2_	3,12	0,25	±	0,07	0,20	±	0,00	0,17	±	0,03
***Creatine***	Cr	CH_3_	3,04	0,22	±	0,16	0,35	±	0,01	0,38	±	0,01
***Ethanol/ Hydroxybutyrate***	EtOH/HB	CH_3_	1,21	0,31	±	0,11	0,62	±	0,02	0,22	±	0,01
***Formate***	For	CH	8,52	0,53	±	0,21	0,41	±	0,06	0,29	±	0,09
***Glutamate/ Glutamine***	Glu/Gln	CH_2_	2,34	0,85	±	0,30	1,40	±	0,01	1,44	±	0,07
***Histidine***	His	CH	7,74	0,23	±	0,11	0,20	±	0,03	0,18	±	0,04
***Lactate***	Lac	CH	4,14	1,01	±	0,25	3,33	±	0,10	3,25	±	0,25
***Lipids***	Lip	CH_3_	0,8–0,9	0,62	±	0,18	0,59	±	0,00	0,66	±	0,01
***Lysine/Arginine***	Lys/Arg	CH/CH_2_	1,6–1,8	0,49	±	0,15	0,50	±	0,01	0,47	±	0,06
***Phenylalanine***	PheAla	CH_3.5_	7,44	0,16	±	0,07	0,15	±	0,02	0,13	±	0,02
***Prolina***	Pro	CH	4,2	0,44	±	0,16	0,64	±	0,06	0,80	±	0,15
***Threonine***	Thr	CH	4,26	0,32	±	0,13	0,51	±	0,05	0,57	±	0,04
***Tryptophan***	Try	CH_7_	7,56	0,24	±	0,11	0,20	±	0,03	0,15	±	0,04
***Tyrosine***	Tyr	CH_2.6_	6,9	0,13	±	0,11	0,12	±	0,03	0,08	±	0,05
***Valine/ Leucine/ Isoleucine***	Val/Leu/Ile	CH_3_	0,9–1,1	0,30	±	0,09	0,69	±	0,02	0,77	±	0,04
***α-Glucose***	α-Glu	CH(H_1_)	5,28	2,93	±	0,63	0,47	±	0,02	0,42	±	0,01
***β-Glucose***	β-Glu	CH(H_1_)	4,68	3,40	±	0,70	0,73	±	0,15	0,69	±	0,05

**Fig 2:**
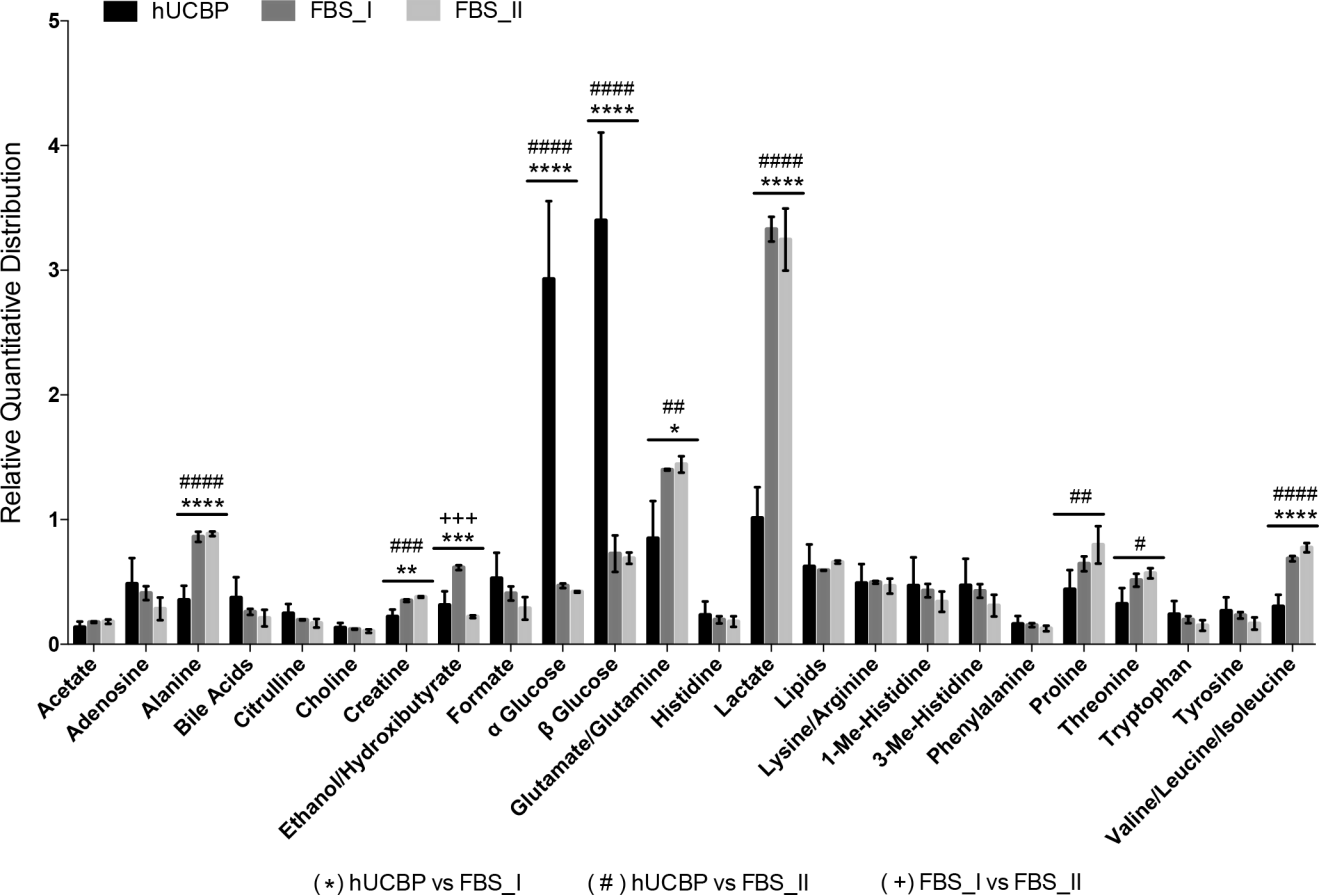
Relative quantitative distribution of the metabolites in hUCBP, FBS_I and FBS_II. Results presented as Mean ± SEM. Symbols reflect differences between hUCBP and FBS_I (*), hUCBP and FBS_II (#), and FBS_I and FBS_II (+), where the numbers of symbols represents the P value (1 symbol: 0.01≤P≤0.05; 2 symbols: 0.001≤P<0.01; 3 symbols: 0.0001≤P<0.001; 4 symbols: P<0.0001).

The presence of essential amino acids useful for protein metabolism and protein synthesis (alanine, arginine, glutamine, glutamate, isoleucine, histidine, leucine, lysine, phenylalanine, proline, tyrosine, and valine), organic acids and derivatives (lactate, β-hidroxibutirate, ethanol, formate, creatine), glucose (α glucose and β glucose), lipids, and nucleotides (adenosine, uridine, guanosine species) were detected in the hUCBP, FBS_I, and FBS_II samples spectra. The relative integral intensity of characteristic signals in ^1^H-NMR spectra of hUCBP samples and FBS samples (both FBS_I and FBS_II) was used to quantify the detected metabolites. The results allowed us to estimate the relative quantitative distribution of metabolites in all studied samples. The metabolic profiles of the FBS samples (both FBS_I and FBS_II) were found to be very similar but demonstrated some differences when compared to analysed hUCBP samples. The average data for the metabolites distribution in hUCBP, FBS_I and FBS_II samples are graphically presented in **Fig 2**.

The results have shown that the major difference between the metabolic profiles of hUCBP and FBS samples is due to the different content of glucose, lactate, glutamate, alanine and branched chain amino acids **(**see **Table 2**for detailed relative quantities). Significantly higher levels of glucose (α glucose and β glucose) and lower levels of lactate were found for the hUCBP than for FBS samples. The analysis of ^1^H-NMR spectra further revealed an increased level of alanine, glutamate, isoleucine, leucine and valine in both commercial FBS (FBS_I and FBS_II) when compared to hUCBP samples. This seems to be the main difference between the amino acids profiles of hUCBP and FBS. Increased levels of alanine and glutamine can be identified in the commercial FBS sera. Apart from the amino acids discussed above, all hUCBP samples were found to have similar or slightly higher contents of arginine, citrulline, choline, histidine, 1(3)-methyl-histidine, lysine, phenylalanine, tryptophan and tyrosine, as well as of nucleotides (δ = 8.4–7.8 ppm), lipids (δ = 0.5–0.9; 1.3; 5.3 ppm), choline and formate when compared to the FBS samples analysed.

#### 3.2.2. PCA of hUCBP samples

**Fig 3:**
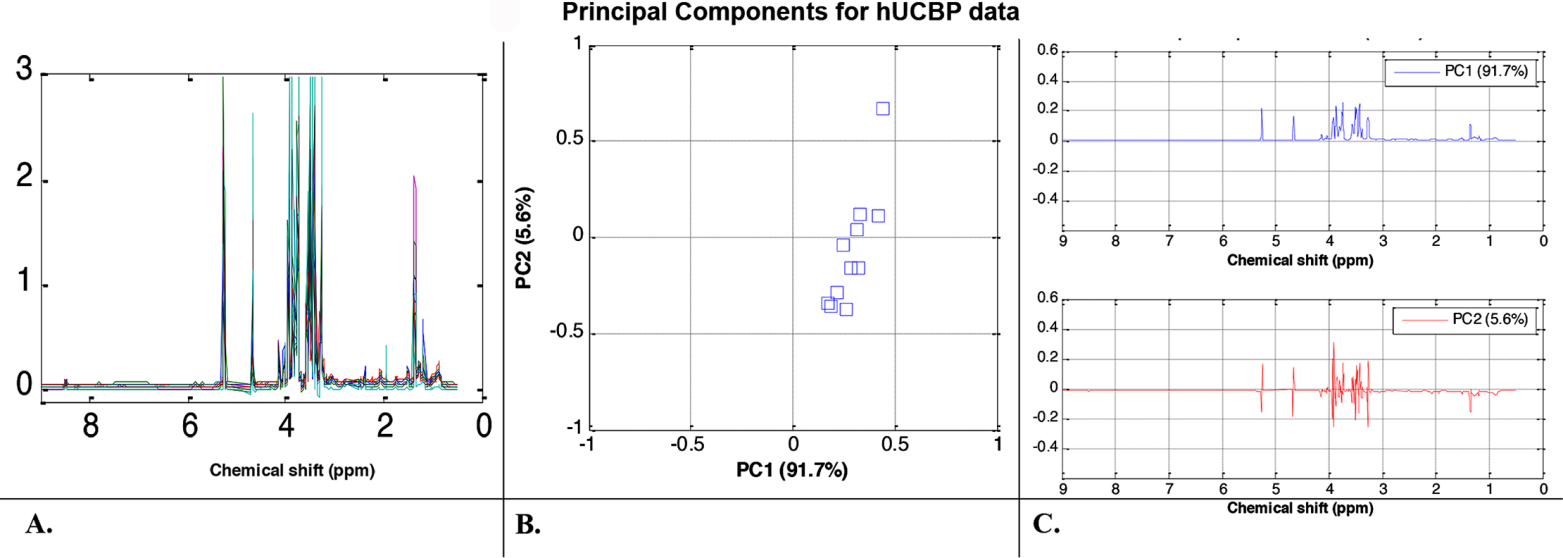
Metabolomic analysis of hUCBP samples. (A) ^1^H-NMR spectral trajectories of the hUCBP samples; (B) 2D (PC1 vs. PC2) PCA score plot shows that hUCBP data are well clustered (i.e. there is high similarity between the donor samples) with exception of one donor (considered as an outlier) and (C) corresponding loadings plots illustrate that sugars and lactate content are the main metabolites responsible for the divergence between the hUCBP samples.

PCA was used to classify the NMR spectral data and revealed factors contributing to the variability between the three groups of data sets. The NMR data in the spectral area between 0.5 and 9.0 ppm was analysed with the water and citric acid spectral regions always excluded prior to PCA analysis and provided as input to the PCA algorithm. The algorithms extracts are the so called Principal Components (PC) that explain data similarity and variability. The hUCBP was first analysed independently of the FBS samples. The ^1^H-NMR spectral data of all hUCBP samples (normalized by the total sum of the integral intensities) are plotted in **Fig 3A**. The observed variations are confirmed by the 2D (PC1 vs. PC2) score plot in **Fig 3B**, where the hUCBP samples are clearly scattered and one typical outlier sample is also detected. Note that the first two PCs accumulated more than 97% of data variance (PC1 has 91.7% and PC2 has 5.9% amount of variance), therefore they are sufficient features to represent the data. The corresponding loadings plots for PC1 and PC2 in **Fig 3C** illustrate that sugars and lactate content are the main metabolites responsible for the divergence between the hUCBP samples.

#### 3.2.3. PCA of hUCBP and FBS samples (FBS_I and FBS_II)

**Fig 4:**
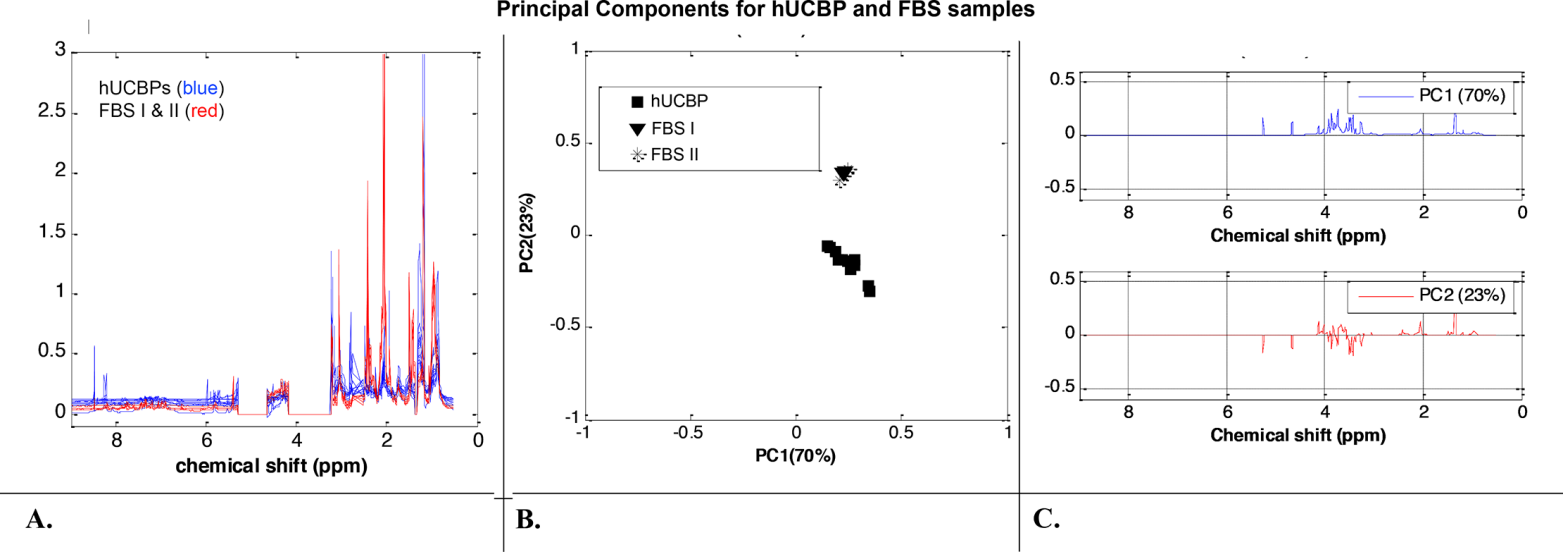
Metabolomic analysis of hUCBP and FBS samples. (A) ^1^H-NMR spectral trajectories of the hUCBP samples (blue colour) and FBS (FBS_I and FBS_II samples) (red colour) groups; (B) 2D (PC1 vs. PC2) PCA score plot shows close similarity of FBS_I and FBS_II data (a well-defined cluster with overlapping samples) and a separation from the hUCBP data and (C) corresponding loadings plots illustrate that glucose, lactate and acetates are the main metabolites contributing to the dissimilarity between the human plasma (hUCBP) and bovine sera (FBS_I and FBS_II) samples. Loading plots around zero line suggest close behaviour of the metabolites profiles in the spectral region from 9.0 ppm to 5.3 ppm of the three groups of samples (hUCBP, FBS_I and FBS_II samples).

Statistical analysis was further applied to the ^1^H-NMR spectra of all samples in order to identify the source of variability between the metabolic profiles of hUCBP, FBS_I and FBS_II. The ^1^H-NMR spectral trajectories of the hUCBP samples (blue colour) and FBS samples (red colour) groups are plotted in **Fig 4A**. Visual data inspection shows the higher levels of glucose but lower of lactate, ethanol, acetates, and several amino acids (alanine, glutamate, valine, leucine, and isoleucine) in the hUCBP when compared to the FBS samples. A matrix with 19 columns (correspond to the samples) over the spectral area between 0.5 and 9.0 ppm with the water and citric acid spectral regions excluded was provided as input to the PCA algorithm. The 2D (PC1 vs. PC2) PCA score plot is depicted in **Fig 4B**. The results illustrated the close similarity of FBS_I and FBS_II data sets (a well-defined cluster with overlapping samples) and revealed their separation from the hUCBP data (a scattered cluster) particularly with respect to PC2. PC1 contains 70% and PC2 23% of data variance, thus these two principal components are sufficient to represent the data information content. The corresponding loadings plots **(Fig 4C)** provide evidence that glucose, lactate and acetates are the dominant metabolites contributing to the dissimilarity between the human plasma (hUCBP) and bovine sera (FBS_I and FBS_II) data sets. Loading plots around zero line suggest close behaviour of the metabolites profiles in the spectral region from 9.0 ppm to 5.3 ppm of the three groups of samples (hUCBP, FBS_I and FBS_II samples). These results coincide with those obtained from the ^1^H-NMR spectral analysis of the samples.

#### 3.2.4. PCA of hUCBP samples and FBS samples (FBS_I and FBS_II) excluding glucose and lactate spectral areas

**Fig 5:**
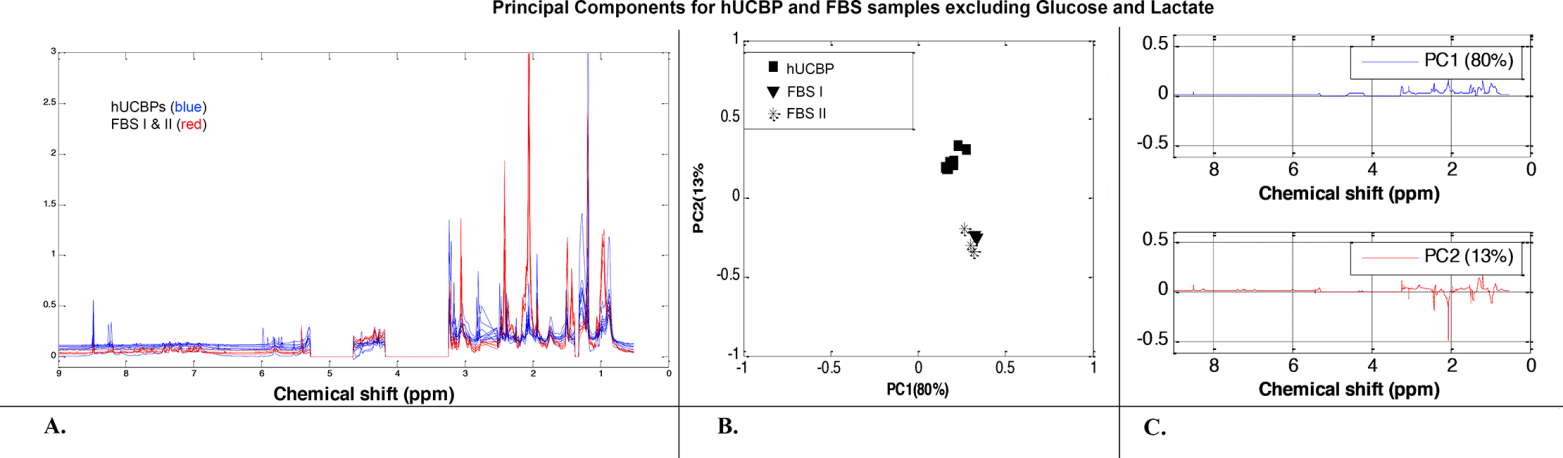
Metabolomic analysis of hUCBP and FBS samples excluding glucose and lactate spectral areas. (A) The ^1^H-NMR spectral trajectories; (B) the PCA score plots shows that the hUCBP samples (a well-defined cluster) and the FBS samples (also a well-defined cluster) are less divergent now (they all have a very close PC1 score) and (C) respective loading plots illustrate that the main differences between the hUCBP and FBS data are due to different concentrations of particular amino acids (alanine, glutamate, isoleucine, leucine, and valine) and organic acids and derivatives (N-acetyl residues, 3- hydroxybutyric acid, and creatine) in the spectral region from 3.3 ppm to 0.5 ppm. For spectral area between 9.0 ppm to 3.3 ppm (with excluded glucose) the metabolites profiles of the three groups (hUCBP, FBS_I and FBS_II samples) are very similar.

In order to assess intrinsic variations in the content of proteinogenic amino acids, lipids and organic acids and derivatives between hUCBP, FBS_I and FBS_II samples, the same PCA analysis was applied on the ^1^H-NMR data after exclusion of glucose (5.28 ppm—4.65 ppm; 4.17 ppm—3.24 ppm) and lactate (1.37 ppm—1.32 ppm) spectral areas. The ^1^H-NMR spectral trajectories are plotted in **Fig 5A** and the PCA score plot is shown in **Fig 5B**. The hUCBP samples analysis was less scattered, while the FBS_I and FBS_II samples continued to behave as a well-shaped cluster. This observation suggests that glucose and lactate concentrations significantly vary between the hUCBP samples of different donors and their exclusion increased the metabolite profiles proximity. The hUCBP and FBS (FBS_I and FBS_II) samples clusters diverge mainly in the PC2 score (representing only 13% of data variance) and have a very close PC1 score (representing 80% of data variance). The respective loading plots (**Fig 5C**) confirm these results. They illustrate that the main differences between the hUCBP and FBS data are due to different concentrations of particular amino acids (alanine, glutamate, isoleucine, leucine, and valine) and organic acids and derivatives (N-acetyl residues, 3-hydroxybutyric acid, and creatine) in the spectral region from 3.3 ppm to 0.5 ppm. From the spectral intervals where the loading plots are close to zero, i.e. from 9.0 ppm to 3.3 ppm, with the glucose spectral area excluded, it can be concluded that the metabolites profiles of the three groups (hUCBP, FBS_I and FBS_II samples) are similar.

**Fig 6:**
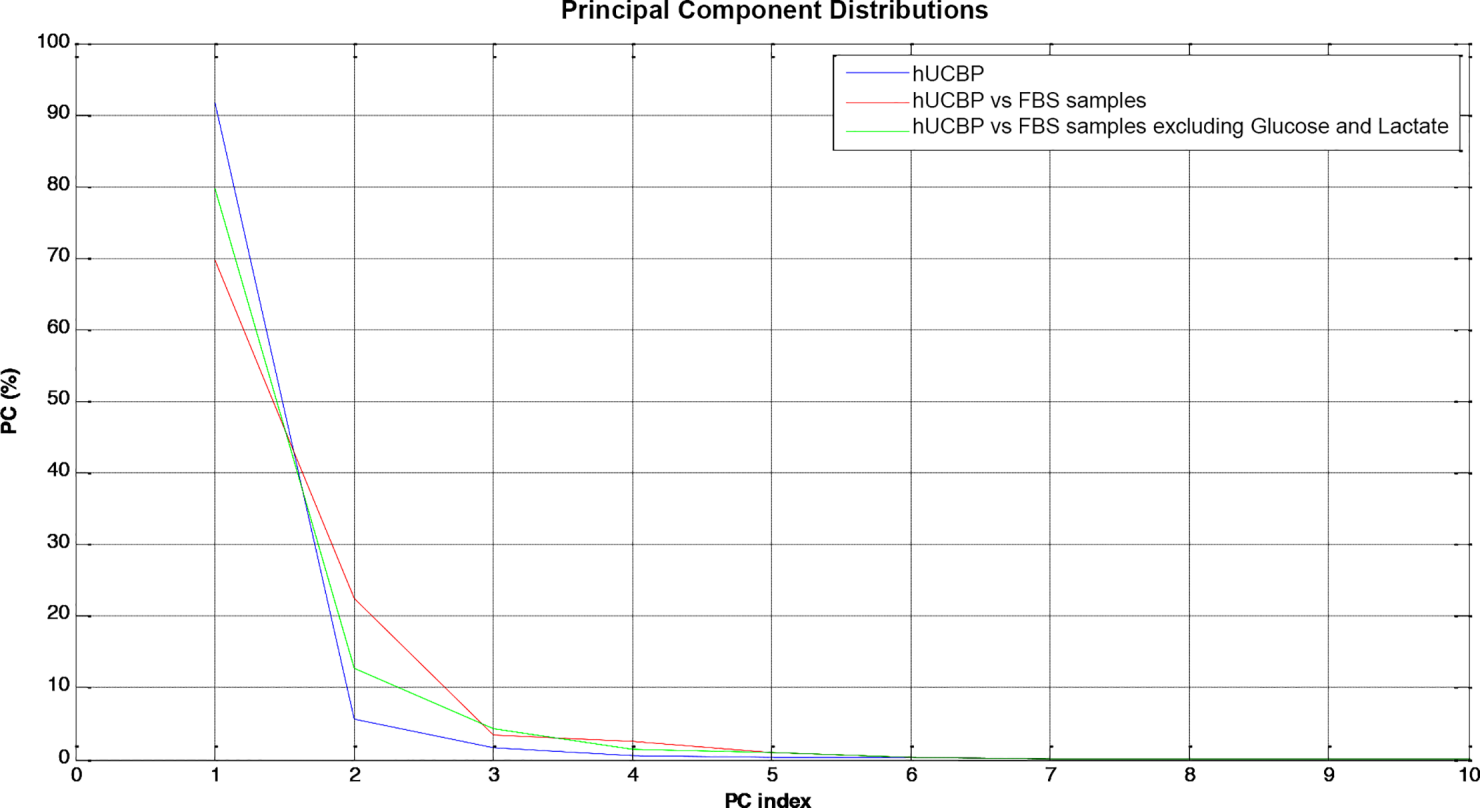
PC contributions for data representation.

PCA analysis was performed in three different scenarios based only on the first two Principal Components (PC). **Fig 6**illustrates that PC1 and PC2 accumulate more than 90% of data variability and therefore their statistical significance is reliable.

### 3.3. Part II: *In vitro* validation

#### 3.3.1. Cell viability assessment

The application of hUCBP as a culture medium supplement for *in vitro* culture and expansion of hMSCs was evaluated using UC-MSCs and DPSCs. Fully characterised cell lots were used for the purpose. PrestoBlue^®^ viability test was undertaken at determined time points and the supernatant absorbance was measured, normalized and corrected according to manufacturers’ instructions. Corrected absorbance values are presented in **S2 Table,** in Supporting information.

**Fig 7:**
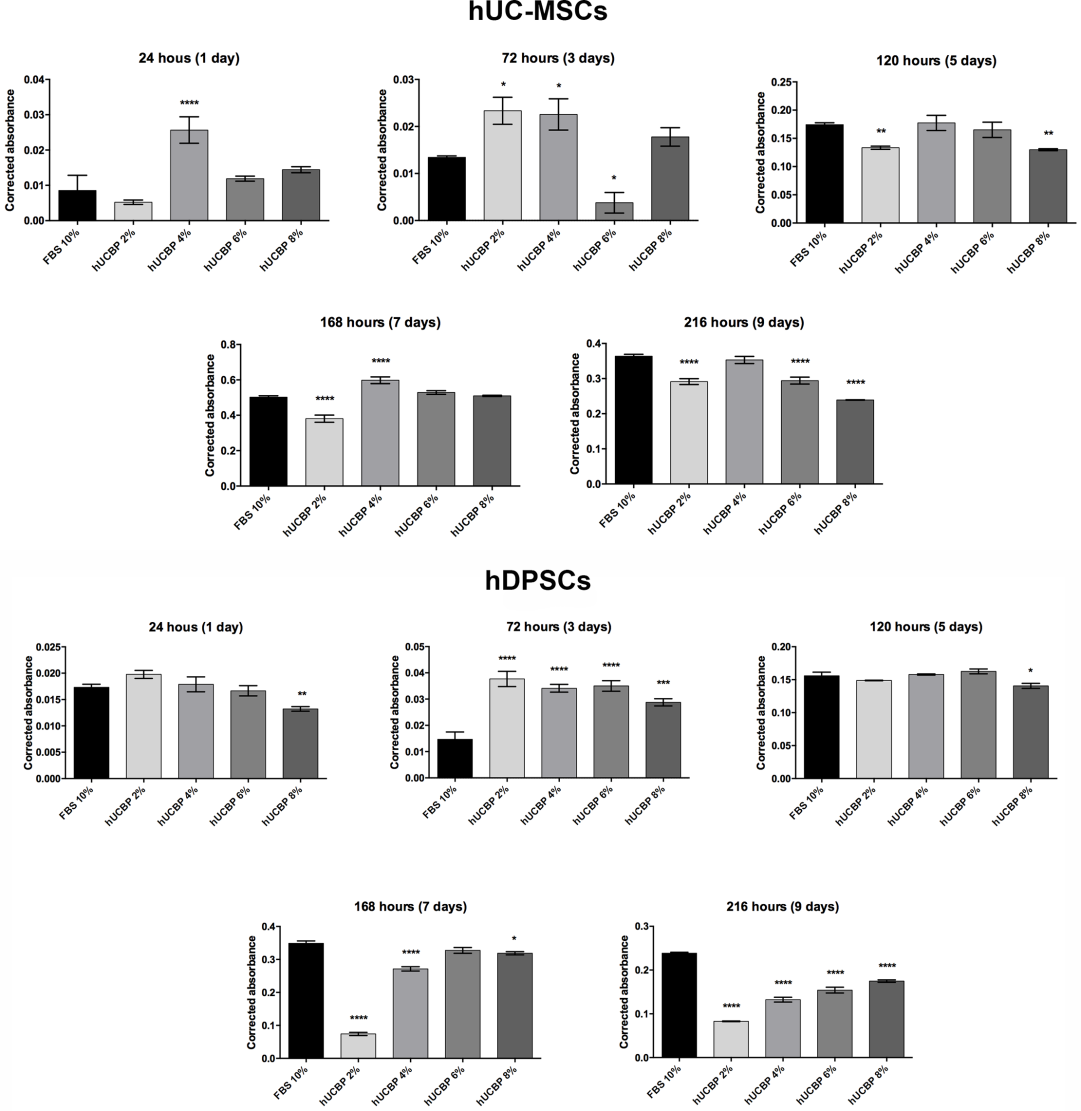
Corrected absorbance readings hUC-MSCs and hDPSCs viability. (A) Corrected absorbance at each assessed time point of the hUC-MSCs cultured in 10% FBS_II, hUCBP 4%, 6% and 8%. (B) Corrected absorbance at each assessed time point of the hDPSCs cultured in 10% FBS_II, hUCBP 4%, 6% and 8%. Values are presented as Mean ± SEM. Statistically differences were analysed for each hUCBP test samples *versus* control (10% FBS_II supplemented medium) and presented with one to four symbol (*) (*: 0.01≤P≤0.05; **: 0.001≤P<0.01; ***: 0.0001≤P<0.001; ****: P<0.0001).

The FBS_II supplemented samples were set as the reference control for the behaviour of hUCBP test samples. Obtained results are graphically presented in **Fig 7**and absorbance data is available in **S2 Table**, in Supporting information.

At 24 hours, UC-MSCs cultures with hUCBP at 4% depicted significantly superior measurements concerning % of cell viability and absorbance, when compared to 10% FBS_II, corresponding to a 200.02% increase over the FBS_II control at that time point. DPSCs presented similar behaviour in most of the tested groups, with no significant differences from the FBS_II control. Here, only the hUCBP 8% group presented inferior measurements (76.28% of cell viability. At 72 hours in culture, UC-MSCs in both hUCBP 2% and hUCBP 4% raised the absorbance to 73.59% and 67.86% over the matched control, respectively. In the DPSCs set, all groups performed superiorly, to the FBS control. In the following time point of 120 hours, no statistical differences were found between the tested groups, for hMSCs from both sources. Maximum measurements were obtained at 168 hours in culture with UC-MSCs at hUCBP 6% and hUCBP 8%, matching 10% FBS_II. On the other hand, hUCBP 4% remained superior to 10% FBS_II control. In the DPSCs, only hUCBP 6% performed comparably to 10% FBS_II, with all other tested groups presenting inferior corrected absorbances.

Both hMSCs types reached the plateau before 216 hours in culture, and hence, metabolic rates (and resazurin reduction) arrested. In all cell culture groups, including the 10% FBS_II, a decrease of the corrected absorbance measurements was observed. Only UC-MSCs cultured in hUCBP 4% supplemented medium sustained activity matching the control.

Absorbance intensity is correlated to the metabolic activity of cells and not directly to their number, but superior number of cells relate to increased viability readings. Retrieved cells numbers and viability (total cell numbers and life cell numbers recovered) by Trypan blue exclusion assay at 24 hours (1day), 72 hours (3 days), 120 hours (5 days), 168 hours (7 days), and 216 hours (9 days) are presented in **Table 3.**

**Table 3:** Retrieved cell numbers (x 10^3^) assessed by Trypan Blue exclusion dye assay of hMSCs (UC-MSCs and DPSCs), in the presence of supplemented medium with FBS_II or variable concentrations of hUCBP after 24 hours (1day), 72 hours (3 days), 120 hours (5 days), 168 hours (7 days), and 216 hours (9 days) incubation. Results presented as Mean ± SEM.

***Retrieved Cells*** ***(10***^***3***^***)***	***UC-MSCs***														
	***FBS 10%***			***hUCBP 2%***			***hUCBP 4%***			***hUCBP 6%***			***hUCBP 8%***		
***24 h (1 day)***	4,23	±	6,35	4,46	±	1,64	6,28	±	2,76	5,97	±	1,08	5,50	±	0,97
***72 h (3 days)***	11,09	±	0,73	19,94	±	7,36	20,70	±	9,17	13,82	±	24,11	16,24	±	5,40
***120 h (5 days)***	94,92	±	5,40	83,54	±	5,46	108,48	±	24,60	118,41	±	28,99	98,87	±	3,60
***168 h (7 days)***	210,17	±	11,24	173,46	±	28,12	229,63	±	23,09	221,25	±	16,71	174,57	±	3,11
***216 h (9 days)***	200,00	±	14,98	166,00	±	11,53	216,00	±	23,69	211,00	±	12,53	182,00	±	3,00
	***DPSCs***														
	***FBS 10%***			***hUCBP 2%***			***hUCBP 4%***			***hUCBP 6%***			***hUCBP 8%***		
***24 h (1 day)***	18,16	±	1,82	24,82	±	2,86	25,82	±	6,14	24,96	±	4,37	16,95	±	1,67
***72 h (3 days)***	39,96	±	23,12	47,26	±	10,85	49,23	±	6,39	52,42	±	9,14	36,88	±	5,28
***120 h (5 days)***	163,13	±	17,46	112,09	±	0,96	227,88	±	3,98	243,48	±	16,68	180,14	±	14,66
***168 h (7 days)***	255,78	±	16,77	110,72	±	22,56	215,30	±	15,45	245,15	±	20,21	204,28	±	9,18
***216 h (9 days)***	250,00	±	7,23	104,00	±	10,21	191,00	±	11,59	231,00	±	7,51	224,00	±	10,82

#### 3.3.2. Senescence and apoptosis

**Fig 8:**
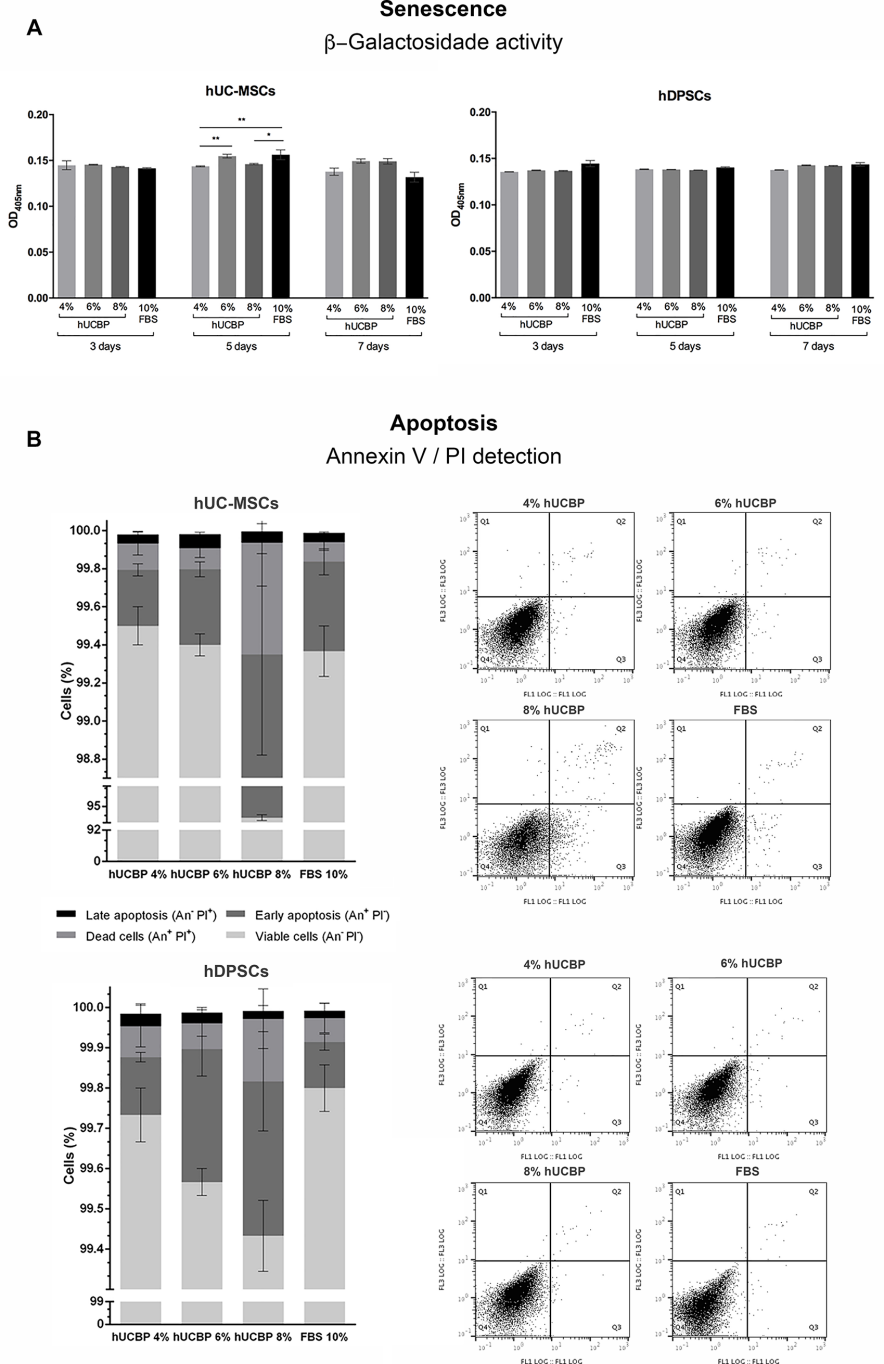
**Senescence and Apoptosis:** A) Senescence - β-Galactosidase activity (OD_405nm_) on UC-MSCs and DPSCs at 3, 5 and 7 days. Results Presented as Mean ± SEM. Significance of the results is indicated according to P values with one, two, three or four of the symbols (*) corresponding to 0.01≤P<0.05; 0.001≤P< 0.01; 0.0001≤P<0.001 and P<0.0001, respectively; B) Apoptosis: Annexin-V/ PI detection of UC-MSCs (upper panel) and DPSCs (lower panel). Results Presented as Mean ± SEM. Dot Plots of the correspondent data are presented at the right side of the panels. Top left quadrants match annexin V negative and PI positive cells; Top right quadrants correspond to dead cells that express annexin V and PI positive; Bottom right quadrants pairs with apoptotic cells that express annexin V positive and PI negative; and for last, bottom left quadrants, viable cells that express neither annexin V or PI.

The assessment of β-Galactosidase activity as an indicator of senescent decline of hMSCs’ populations cultures in hUCBP supplemented media revealed no striking differences when compared to FBS supplemented control media, for both assayed populations (**Fig 8A** and **S3 Table,** in Supporting information). Exception was noted for cultured UC-MSCs, at 5 days in culture, where FBS supplemented groups displayed increased β-Galactosidase activity when compared to 4 and 8% hUCBP supplemented groups.

Annexin-V/ PI assay was employed for the detection and definition of apoptosis in cultured cells. Early apoptosis is characterized by plasma membrane reorganization, detected by positive staining for Annexin V, while later stage of apoptosis presents membrane damage, therefore PI can bind to DNA in cytoplasm resulting in positive staining for both Annexin V and PI [53]. Cells cultured under hUCBP or FBS supplementation revealed no significant differences in Annexin-V or PI staining, with exception to hUC-MSCs cultured in hUCBP 8% supplementation (*), which presented increased early apoptosis events, and decreased overall cell viability over the remaining UC-MSCs’ groups (% Viable cells (An^-^ PI^-^): 99,50 ± 0,06; 99,40 ± 0,06; 92,93 ± 0,52; 99,37 ± 0,13, and % Early apoptosis (An^+^PI^-^): 0,29 ± 0,03; 0,40 ± 0,04; 6,42 ± 0,53; 0,47 ± 0,07, for 4%, 6%, 8% hUCBP and 10% FBS supplemented media) (**Fig 8B** and **S4 Table** in Supporting information).

#### 3.3.3. MSCs’ Phenotype identity

**Fig 9:**
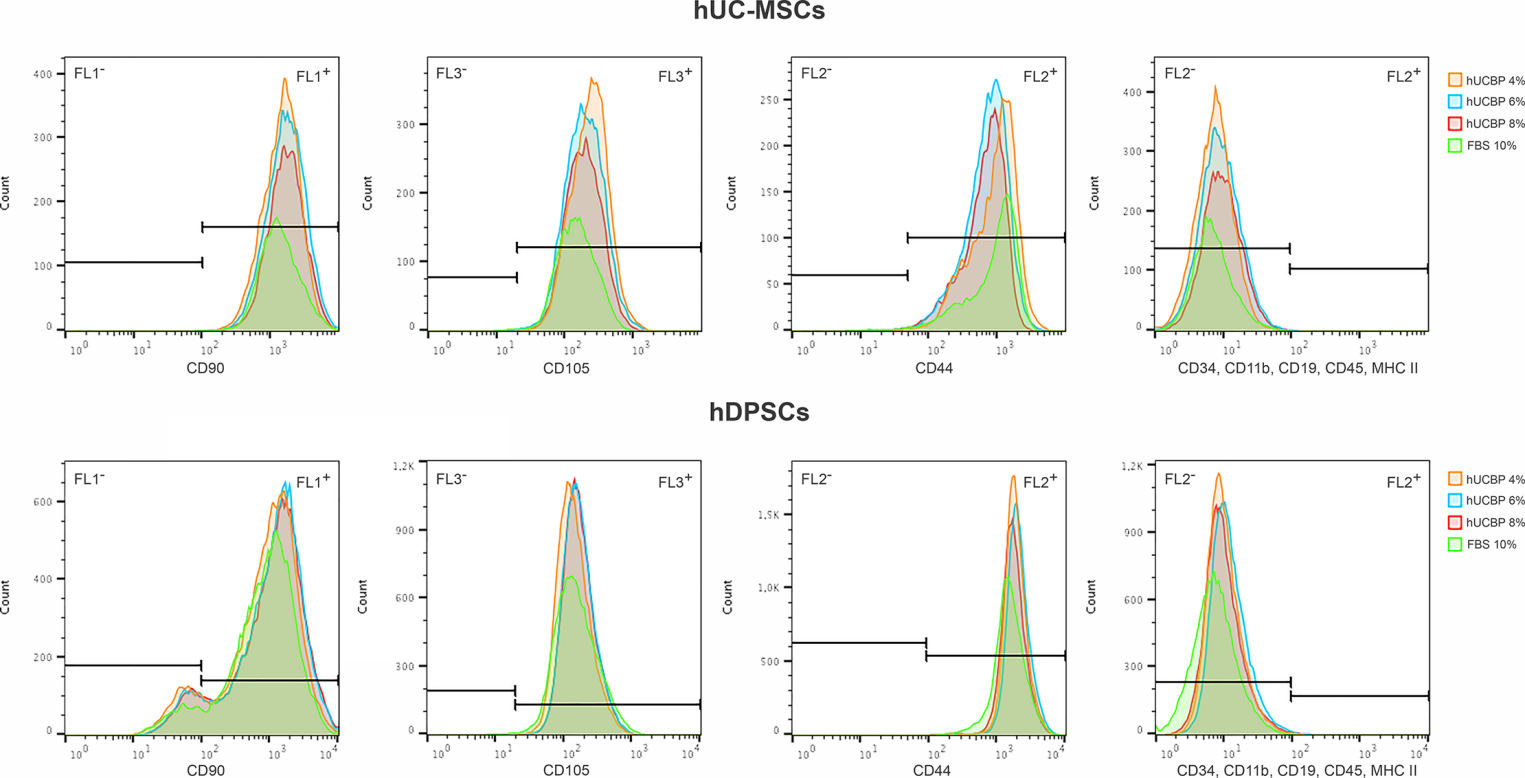
Surface marker expression for MSCs’ identity of hUC-MSCs and hDPSCs, assessed by Flow cytometry.

The surface marker profiles of UC-MSCs and DPSCs cultured in either hUCBP or FBS supplemented media were assessed though Flow Cytometry. MSCs populations presented characteristic MSCs phenotypical markers in all culturing conditions (**Fig 9**and **Table 4**), and although some slight differences are noted, populations remain within the ≥ 92% positive population for CD90, CD105 and CD44, and ≤ 2% negative staining for CD34, CD11b, CD19, CD45 and MHC II. Remarks are to be made regarding the CD90 expression by hDPSCs, which presented only 87–91% positive population, contrasting with 99–100% expressing population of hUC-MSCs.

**Table 4:** Surface marker profiles of hUC-MSCs and hDPSCs cultured in either hUCBP or FBS supplemented media, assessed though Flow Cytometry. *NEG+ cocktail corresponds to anti-CD34; -CD11b; -CD19; -CD45; and -MHC II antibodies.

***Surface Markers***	***hUC-MSCs***											
	***hUCBP 4%***			***hUCBP 6%***			***hUCBP 8%***			***FBS 10%***		
***CD90***^***+***^	99,90	±	0,00	99,83	±	0,03	99,87	±	0,03	99,23	±	0,12
***CD105***^***+***^	99,97	±	0,03	99,77	±	0,03	99,87	±	0,03	99,07	±	0,09
***CD44***^***+***^	99,47	±	0,09	99,40	±	0,15	99,27	±	0,20	95,70	±	1,65
***NEG***^+^*	0,05	±	0,01	0,14	±	0,04	0,25	±	0,14	0,13	±	0,08
	***hDPSCs***											
	***hUCBP 4%***			***hUCBP 6%***			***hUCBP 8%***			***FBS 10%***		
***CD90***^***+***^	90,23	±	0,45	90,70	±	0,15	90,67	±	0,07	90,50	±	0,35
***CD105***^***+***^	99,90	±	0,00	100,00	±	0,00	99,97	±	0,03	99,30	±	0,06
***CD44***^***+***^	99,97	±	0,03	99,80	±	0,00	99,83	±	0,03	99,80	±	0,00
***NEG***^+^*	0,37	±	0,03	0,52	±	0,05	0,52	±	0,09	0,13	±	0,04

#### 3.3.4. *Gene expression*

**Fig 10:**
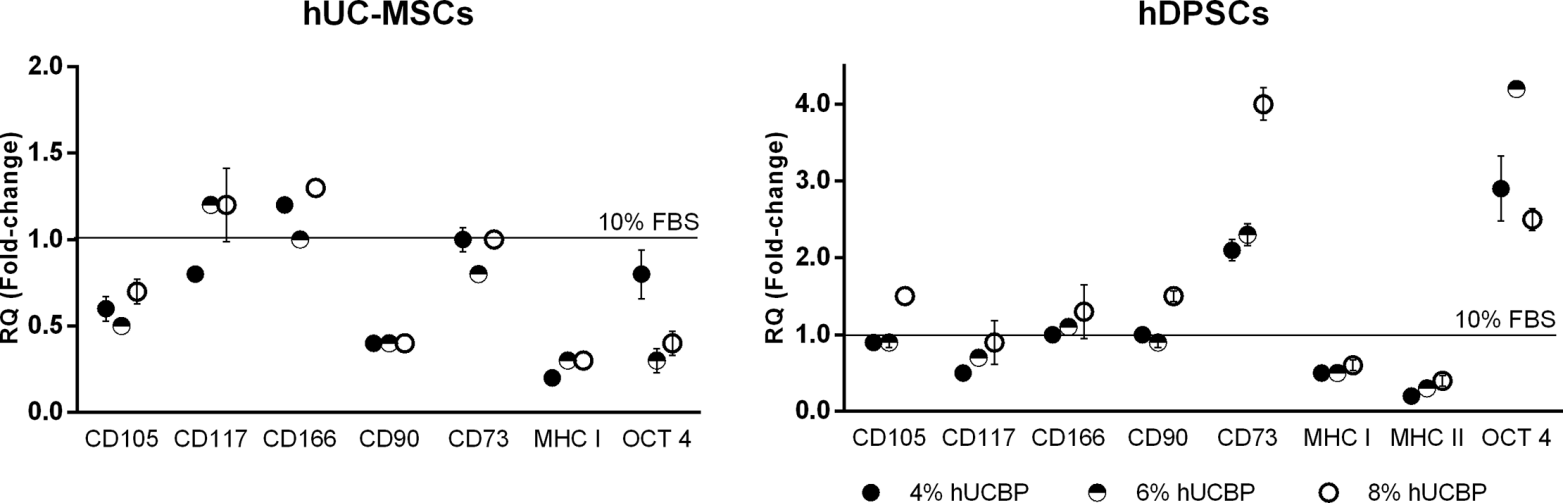
Quantitative RT-PCR of UC-MSCs and DPSCs cultured in hUCBP or FBS supplemented media. RQ: relative quantification (fold change compared to the FBS 10% group), in mean fold change ± SEM.

Gene expression was performed through RT-qPCR analysis. Total RNA was successfully extracted from cultured hMSCs (**S5 Table,** in Supporting information) and specific genes’ expression assessed, after 5 days in specific culturing media conditions. CD34 and Sox2 were undetected in any of the samples from hUCBP or FBS cultured cells, in either population. Strong expression (<29 Cq) of CD105 and CD166, and moderate expression (30–35 Cq) of CD115 were detected in all assayed groups, and no change in expression was observed in differently supplemented UC-MSCs and DPSCs cultures. Particular changes in fold-expression of specific genes was identified in hMSCs from each source (**Fig 10**and **S6 Table,** in Supporting information). In UC-MSCs, CD90 and MHC class I were down-regulated in all hUCBP supplemented groups (although maintaining a strong expression), and moderately expressed OCT-4 was down-regulated in the highest % supplementation groups (6 and 8%). Expression of MHC class II sequence was not detected in 10% FBS supplemented cultures, while it became weakly expressed (>35 Cq) in hUCBP supplemented groups. In DPSCs, strong and moderate expression of CD73 and OCT-4, respectively, was observed in 10% FBS and up-regulated in hUCBP supplementation groups. Contrarily, weak expression of MHC class II was detected in 10% FBS supplemented hDPSCs, that tended to attenuate (down-regulate) in hUCBP supplemented groups.

#### 3.3.5. Multilineage differentiation

**Fig 11:**
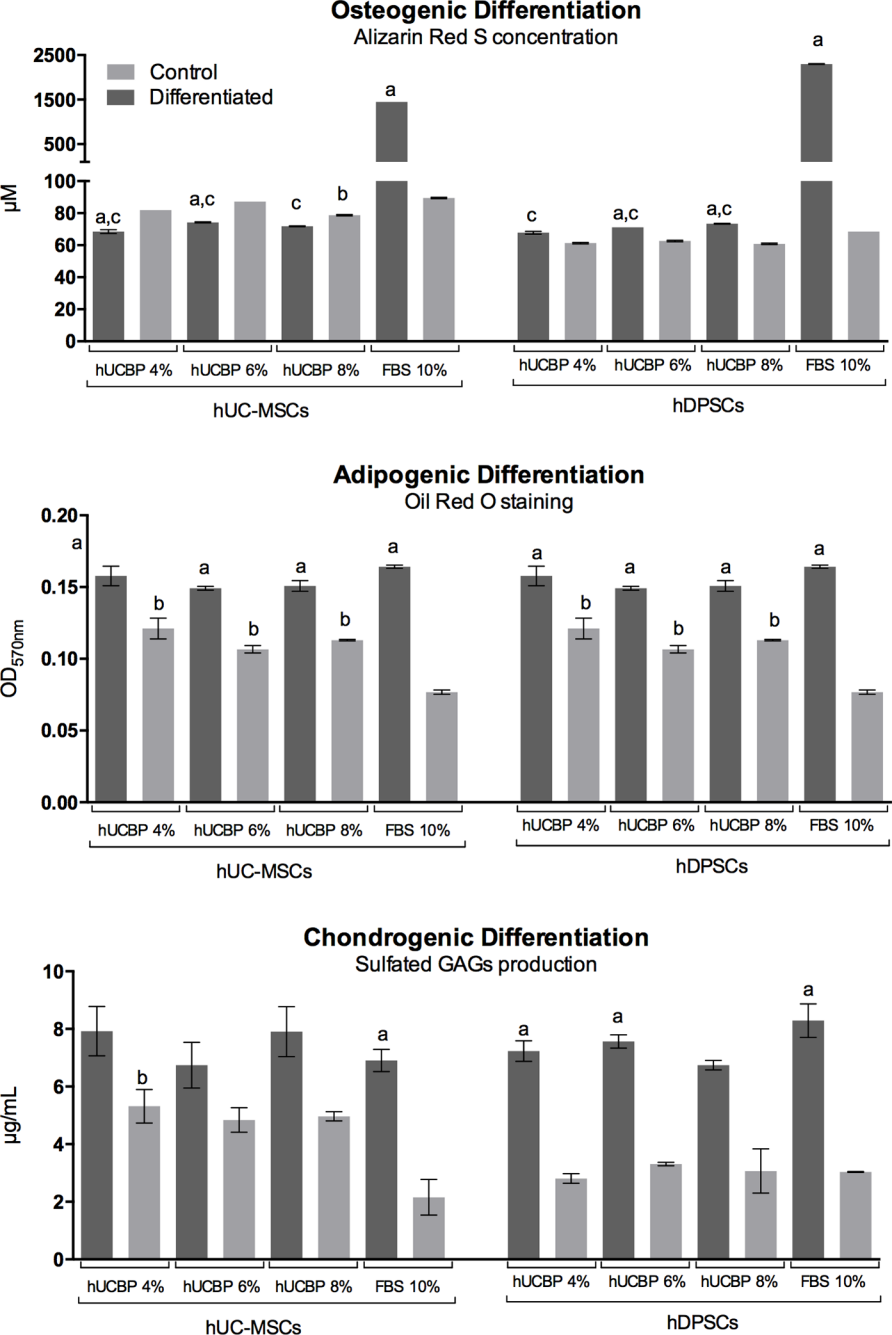
**Multilineage differentiation:** A) Osteogenic differentiation: Alizarin Red S concentration (μM) after 21 days; B) Adipogenic differentiation: Oil Red O (OD_570nm_) after 14 days; and C) Chondrogenic differentiation: Sulfated GAGs production (μg/mL) after 14 days, assessed by Blyscan™ Glycosaminoglycan Assay (Biocolor, UK). Control: Undifferentiated control; Results Presented as Mean ± SEM. a: significantly different from undifferentiated group within the same media condition and cellular population; b: undifferentiated control group significantly different from undifferentiated FBS 10% group, within the cellular population; c: differentiated group significantly different from differentiated FBS 10%, within the cellular population.

Osteogenic differentiation—Macroscopic observation revealed strong Alizarin Red S staining in the FBS supplemented Osteogenic media groups, in both UC-MSCs and DPSCs. All Undifferentiated control groups presented weak ARS staining. Whereas, in all hUCBP supplemented Osteo-differentiation groups, in both cell types, weak ARS staining was observed, not empirically distinguishable from respective control groups. Observation suggest an effective osteo-differentiation and mineral matrix deposition by hMSCs cultures in Osteogenic media containing FBS, while hUCBP supplemented groups did not differentiate towards the intended lineage, after 21 days in specific culture (data not shown). ARS binding quantification confirmed these observations (**Fig 11A**). Some of the differentiating groups presented no statistical difference towards the respective undifferentiated controls (**S7 and S8 Tables,** in Supporting information). Furthermore, the semi-quantitative analysis points towards an increased efficiency in the Osteogenic differentiation of DPSCs when compare to UC-MSCs, under the same differentiating conditions (2299.31 ± 7.35 μM and 1449.57 ± 0.00 μM of ARS, respectively).

**Adipogenic differentiation**—Microscopic observation of ORO stained cells evidenced comparable lipid droplets accumulation in the FBS supplemented Adipogenic media groups, in both UC-MSCs and DPSCs. Controls presented occasional lipid droplets accumulation. All hUCBP supplemented Adipo-differentiation groups presented similar differentiation pattern to FBS supplemented groups, amongst all groups of UC-MSCs and DPSCs. Cellular populations maintained in hUCBP presented lipid droplets accumulation in the cytoplasm (data not shown). Spectrophotometric measurement of ORO staining indicated no significant difference between different supplementation levels and/or FBS, within each cell type. However, control groups maintained in hUCBP indicate significantly superior ORO staining over FBS supplemented controls (**Fig 11B** and **S9 and S10 Tables,** in Supporting information).

**Chondrogenic differentiation**—Groups cultured in Chondrogenic Differentiation media all presented marked light blue staining under macroscopic and microscopic observation, indicating proteoglycan deposition. Control groups from DPSCs in UCBP and FBS remained unstained, indicating no differentiation, as did UC-MSCs in FBS supplemented media. Control groups of UC-MSCs maintained in hUCBP for the assay period presented mild Alcian blue staining and aggregation, suggesting formation of chondrogenic pellets and deposition of proteoglycan rich matrix (data not shown). UC-MSCs and DPSCs maintained in FBS supplemented control media presented no difference in terms of GAGs production, and cells transitioned to differentiating media after 3 days showed increased GAGs production, demonstrating differentiation towards a chondrogenic phenotype, with no significant differences between cell types **(Fig 11C** and **S11 and S12 Tables,** in Supporting information). Cells expanded in hUCBP at different concentrations and transitioned to Chondrogenic differentiation media, displayed comparable GAGs production. In the DPSCs, cell maintained in hUCBP for the 14 days of the differentiation period did not differ from the ones maintained in FBS 10%. Undifferentiated controls of UC-MSCs’ maintained in the hUCBP supplemented media demonstrated a tendency to produce increased GAGs, when compared to controls, with mean values approaching differentiated groups, and not being significantly different from these.

## 4. Discussion and conclusions

In light of the observations from this study, the option of utilizing hUCBP as a potential replacement to FBS supplementation for hMSCs expansion gains further support. The first part of the study focused on the characterization the metabolite composition of hUCBP and its comparison to conventionally utilised FBS, to address its suitability as a culture medium supplement and FBS substituent. In primary samples analysis, individual variations were observed concerning the CD34^+^/CD45^+^ and WBC cells counting, where different donors contribute to a wide range of values. One of the samples was detected as contaminated by anaerobic agent *Actinomyces meyeri* (hUCBP#5), which reinforces the need for thorough quality control of hUCBP samples before usage for cellular therapies preparation. To the authors’ knowledge, this is the first extensive comparative study on the metabolomic profile of FBS and hUCBP. Previous published work contributed to a deeper understanding on the growth factor, cytokine and chemokines content of hUCBP, suggesting that it could play a relevant role in advanced therapies development [8].

Within hUCBP samples, glucose and lactate are the major contributors to inter-samples metabolite composition variations. Overall, these observations suggest that, similarly to FBS supplements, the hUCBP are not homogeneous, and present great individual variability, thus requiring to be primarily homogenized before being used in cell-based therapies preparation [41–43]. Hence, the composition of pooled hUCBP batches is well suited for standardization, to which this work aims to contribute with relevant data in terms of individual samples compositions. This will aid in the definition of reference ranges of key components for the approval and release of representative batches, targeting at the reduction of inter-batch variability.

In addition to the detailed definition of individual samples and pooled plasma batch composition as well as the standardization of reference ranges, other concerns must be observed regarding hUCBP quality and safety analysis. Strict control of potential infectious diseases transmission is required at the various steps of the process. One limitation from the necessity of pooling of multiple donor samples is the increased potential risks of infection via donors' hUCBP. To counterbalance this risk, control step reinforcement is advised when aiming at therapeutic applications, with further monitoring of individual samples upon collection and following homogenization/ pooling process.

Comparatively, metabolite profiles of both supplements present distinct features, as demonstrated by NMR spectroscopy and subsequent PCA analysis. The main difference between the metabolic profiles of the two proposed supplements was due to the significantly higher levels of glucose (both α and β glucose) and lower of lactate in the hUCBP. The increased glucose content in hUCBP was attributed to the function of UCB to provide energetic and other metabolic support for the foetus during intrauterine development. The observed variation in the glucose levels throughout hUCBP samples has also been ascribed to the particular diet and health conditions of the different donors, as described by other researchers [41–43]. The presence of anticoagulant citrate phosphate dextrose solution (CPD) [composed of 2.63g of sodium citrate (dehydrate), 0.299 g of citric acid (monohydrate), 2.55g of dextrose (monohydrate) and 0.222g of sodium monobasic bisphosphate (monohydrate)] in the UCB sterile collecting bag might have contributed to the high levels of carbohydrates, as well as of citric acid, detected in ^1^H-NMR spectra of hUCBP. Culturing media usually contain varying concentrations of glucose ranging from 1 to 4.5 g/l (5.6 to 25 mM). Glucose is a central source of energy for hMSCs, and recently isolated cells normally benefit from high glucose during the first 48 hours in culture, as we have observed [12, 54, 55]. However, sustained elevated glucose concentrations may impair cellular functions, increase the intracellular calcium concentration ([Ca^2+^]_i_) and induce apoptosis [12, 47, 54]. It has also been demonstrated that high glucose stimulates the glucose-responsive gene thioredoxin-interacting protein (Txnip) in MSCs and induces MSCs to a pro-apoptotic situation, which reduces their regenerative qualities after chronic but not short-term exposure [55]. Nevertheless, high glucose contents must not always be regarded as deleterious for hMSCs populations, as it appears to greatly improve survival in severe hypoxia situations that are often associated with pathological events of ischemia and stroke [56]. The glucose content of the culture medium is therefore of the utmost importance. Increased levels of lactate are detected in FBS samples. In the haemoderived cellular culture supplements, particularly those collected in perinatal stages, as is the case of FBS and hUCBP, observed lactate levels strictly relate labor process and metabolic acidosis of the newborn, and is an important indicator of intrapartum asphyxia [57]. Lactate is a product of anaerobic metabolism of pyruvate (that mostly derives from supplied glucose), and accumulates in cellular culture supernatant during cell culturing, indicating a coexistence of both aerobic and anaerobic bioenergetic pathways. We have recently focused on the characterization of the metabolic profile of these precise cellular populations, and have verified that small amounts (in the μM range) of lactate are provided by basal culture media, and that this metabolite accumulates steadily for up to 48 hours in the culture media (reaching concentration in the 100–200 μM range) (Unpublished data). Most of the research targeting this topic focuses on lactate production by cultured populations. Fewer references focus on the effects of the lactate on these population, and mostly focus on its effects when accumulated in result of bioenergetic production. Lactate is described to impact on cell proliferation, resulting in inhibition over 20mM lactate in the culture media [58], but little is commented on the effects of low concentration of such metabolite. FBS samples assayed are commercially available and have been successfully employed in the hMSCs culture, as represented by the control groups herein utilized. Metabolomic analysis indicated increased increase relative amounts of lactate in these FBS samples, when compared to the hUCBP. FBS and hUCBP are supplemented to culture media in small percentages, ranging from 2–10% (in the presented experimental work) and hence observed variations in the metabolite content are diluted in the final media formulation. Since lower content in lactate is observed in the hUCBP samples, it is not expected to negatively impact cell performance. Although lactate is recognised to affect MSCs proliferative and differentiation features at high concentrations [59], FBS supplementation is not assigned to provide inhibitory levels of lactate. Both FBS groups show similar metabolite profile that differ from hUCBP samples in their high content of lactate, alanine, glutamine and branched chain amino acids and lower of glucose. Similar or slightly different levels of important proteinogenic amino acids, and of nucleotides, lipids, choline and formate were found in both samples. These proteinogenic amino acids are proteins precursors, and thus play a crucial role in the growth, proliferation and differentiation of hMSCs. The decreased content of alanine and glutamine in the hUCBP samples could be theoretically compensated by adding culture media supplements such as GlutaMAX^TM^ (Thermo Fischer Scientific). This animal-origin free supplement (L-alanyl-L-glutamine) is a dipeptide substitute for L-Glutamine and can be used as a direct substitute for L-Glutamine at equimolar concentrations in mammalian and stem cell culture with minimal or no adaptation [8, 60]. At a cellular level, there are many important functions of amino acids such as glutamine, being essential as precursor for peptide and consequently substrate for protein synthesis [61–63]. Choi and colleagues [64] reported on importance of adequate essential and non-essential amino acids in cell culture, as over supplementation of the later was demonstrated to negatively affect MSCs proliferation. On the other hand, increased levels of essential amino acids, with balanced non-essential amino acids intake, were associated with increased cell proliferation. MSCs’ surface markers expression did not seem to be affected by differential amino acid contents.

Other minor differences in key proteins’ content are observed. Proline, a non-essential amino acid, is overexpressed in FBS_II samples, and has been associated with suppressing reactive oxygen species (ROS), thereby protecting mammalian cells from various stresses [65, 66]. FBS_I showed higher ethanol content in comparison to FBS_II. It has been reported that stem cells are extremely sensitive to alcohol by-products such as acetaldehyde that induces irreversible DNA mutation [67–69], and that it induces adipogenesis, resulting in cell death [70]. In other reports, ethanol exposure interfered with pluripotency marker patterns [68]. Therefore, hUCBP’s lower content in ethanol arises as a beneficial point for the maintenance of the regenerative potential of expanding hMSCs. This particular finding converges with our preliminary experience in hMSCs *in vitro* proliferation and expansion, which supported the choice of FBS_II to further study the comparative effect of hUCBP culture medium supplementation [4–12, 29, 47].

The second part of the study aimed at the validation of hUCBP for the expansion of umbilical cord and dental pulp derived mesenchymal stem cells. Proliferation and viability/metabolic assays demonstrated that hMSCs were able to proliferate in 4%, 6% and 8% hUCBP supplemented culture media, and that metabolic rates observed (determined by Presto Blue day reduction assay) were superior to those of DPSCs and UC-MSCs for up to 5 and 7 days, respectively. At this point, cultured cells reached confluency, eliciting inhibitory signaling within hMSCs populations, and hUCBP supplemented cultures increased metabolic rates arrested. Zhang and colleagues reported that high glucose content in the MSCs culture media (which is the most striking difference between hUCBP and FBS) resulted in impaired cellular proliferation [71]. However, this study referred to glucose concentrations ranging from 11 to 20 mM (1.98 to 3.6 g/L, respectively), which exceeds the maximum concentration provided by hUCBP supplemented media [10% FBS: 4,9mM; 4%hUCBP: 6,6mM; 6%hUCBP: 7,5mM; and 8%hUCBP: 8,6mM], and refers to 14 days of culturing, with no mention of passaging or culture confluency. Another study reports no deleterious effect on MSCs’ proliferation of 20-30mM of glucose, but this parameter was only assessed at 48 hours of culturing [72]. The arrest in proliferation rates observed in our study is directly linked to the confluent state of the cultured populations, which are described to shift to inhibitory intercellular signaling (reflected by metabolic rate decrease), due to close cell-to-cell contact. The same study [71], reported the induction of cellular senescence in the high glucose groups. Cellular senescence of cultured cellular populations reflect cellular aging *in vivo*, and this process is associated (although not solely bound to) β-Galactosidase enzyme expression and activity [73]. We observed that short term culturing (for up to 7 days) of passage 5 hMSCs in hUCBP supplemented media does not elicit increased cellular senescence events, as reported by Chang and colleagues, for the same time of assessment [74]. Again, contradictory observation may be due to the extreme glucose concentrations and culturing periods reported [71]. Similarly, cultured populations were assessed for apoptotic events, though the detection of Annexin-V and PI. Annexin V identification indicates the externalization of the membrane phospholipid phosphatidylserine (PS), which happens in the early stage of apoptosis. The positive of PI indicates access to intracellular genetic material, due to cell membrane compromise, which occurs either in the end stage of apoptosis or in dead cells. Therefore, viable, early apoptotic, late apoptotic and dead cellular populations can be distinguished [53]. We observed that culturing in hUCBP supplemented media resulted in a dose related tendency for increased early apoptosis events (Annexin V^+^ and PI^-^ cells) in both UC-MSCs and DPSCs. Supplementation with 8% hUCBP presented seemingly inferior viable populations, with increased dead cells. These events were particularly evident in UC-MSCs populations. High glucose contents are reported to induce oxidative stress in exposed population, which drive MSCs towards apoptosis, through the activation of intrinsic cellular Caspase pathways [75]. These observations align with the apparent dose dependent increase in early apoptosis observed herein [74].

The classification of specific populations as MSCs is related to the expression of a particular immunophenotype. As such, it was agreed by the scientific community [2] that 95% of the candidate population ought to express CD105, CD73 and CD90, as well as to lack expression (<5% positive) of CD45, CD34, CD14 or CD11b, CD79a or CD19 and MHC class II. Other markers, such as CD44 are also identified in hMSCs populations. Cultured hMSCs populations maintained >95% expression of CD105, CD44 and CD73, while remaining <2% positive for CD34, CD11b, CD19, CD45 and MHC class II. Despite the ISCT’s guidelines for the classification of MSCs populations, surface marker identity is a controversial topic. The obligatory expression of high levels (>95%) of CD90 in addressed populations is not always confirmed, while all other MSCs characteristics are verified, such as tri-lineage differentiation capacity [64, 76]. We observed maximal expression of CD90 in hUC-MSCs, but lower levels are equally reported [77]. The expression levels of CD90 have been related to MSCs their immunosuppressive capabilities, as Campioni and colleagues have disclosed [78]. Interestingly, qRT-PCR revealed that, although remaining strongly expressed (in accordance to external marker expression revealed by Flow Cytometry), CD90 depicted decreasing mRNA expression in hUCBP cultured groups, which has been associated to decreased immunosuppressive capacities [78]. MSCs are also described to express MHC class I and, to a much smaller extent, MHC class II [79]. DPSCs presented weak expression of MHC class II molecules, which attenuated in xeno-free cultured groups. Contrarily, MHC class II was not detected UC-MSCs in FBS supplemented groups, but became apparent in hUCBP cultured groups. These expression up- and down-regulations are smooth, a did not reflect in the membrane expression of the molecule [80]. MSCs are described to express pluripotency genes in resemblance to embryonic stem cells. We confirmed c-kit and OCT-4 expression but were unable to detect Sox-2 mRNA. Similarly to the character and degree of expression of surface markers, pluripotency genes expression provides conflicting reports [81, 82]. Distinct patterns of up- and down-regulation of specific genes in UC-MSCs and DPSCs, along with the distinct responses verified in terms of apoptosis and multilineage differentiation capacity, reinforce that differently sourced MSCs respond differently to environmental cues, whether they are provided by their specific niches or by *ex-vivo* culturing conditions. The replacement of FBS in the specific Osteo-differentiation media presents a deleterious effect, as only minor staining was observed after incubation with ARS (a Ca^2+^ binding dye). Quantification confirmed the inferior uptake in all hUCBP groups, as compared to the FBS supplemented groups. Standard FBS supplemented differentiation efficiency also support reported superior capacity from DPSCs to produce mineralized matrix when compared to UC-MSCs [83]. Expansion of hMSCs in media supplemented with hUCBP does not preclude their capacity to differentiate towards adipogenic phenotypes in either cell type, when compared to FBS supplemented groups. The maintenance of UC-MSCs and DPSCs in hUCBP supplemented media for 14 days induced lipid droplet deposition, which although statistically inferior to cells maintained in differentiation media, was superior to the respective FBS supplemented control. The presence of AGE (advanced glycation products, associated with high glucose levels) in culture media is associated to reduced MSCs’ differentiation capacity [54]. Cramer and colleagues [75], also report impaired osteogenic and chondrogenic potential of MSCs, but observed enhanced adipogenic tendency. Again, maximum concentration provided by hUCBP formulated media remains under the studied ranges reported in literature, but similar effects were already observed in osteogenesis and adipogenesis of UC-MSCs and DPCSs. We do not observe the same inhibition in chondrogenensis. Although high glucose is a marking feature oh hUCBP, other relevant components must be addressed in its composition, such as growth factors, cytokines and chemokines [8]. Preliminary assays demonstrate substantially increased levels of TGF-β 1–3 in hUCBP samples when compared to FBS (7-, 2- and 28-fold increase in TGF-β 1, 2 and 3, respectively, data not shown). TGF-β 1 is a strong modulator of TGFβ receptors expression, and thereof of chondrogenesis of MSCs. Although the effects of high glucose are seemingly deleterious to this differentiation pathway, it is associated with increased TGFβRII expression. The combination of both factors (high concentration of glucose and TGF-βs) may attenuate the reported deleterious effect of glucose. We observed that chondro-differentiation efficiency was unaffected by the expansion in hUCBP supplemented media, and that the maintenance of undifferentiated control groups in hUCBP induced UC-MSCs to form chondrogenic-like pellets and produce GAGs rich matrix. The differential response observed in the assessed cell population could relate to the differentiation aptitude of MSCs derived from particular niches [83].

In conclusion, regardless of the discussed differences, FBS and hUCBP present relevant similarities. This study reveals the existence of essential components (including glucose and proteinogenic amino acids) for the growth and proliferation of hMSCs in both supplement options. These observations suggest that hUCBP could be considered as an alternative to FBS for *in vitro* production of hMSCs for cell-based therapies and for clinical applications. Nevertheless, investigation must deepen regarding the applicability of hUCBP through the entire hMSCs production process, and studies are ongoing as for their suitability for the primary isolation of cells from source tissues, as well as for cryopreservation and final formulations of hMSCs derived therapeutic products. Further, we have observed some of the hUCBP composition on the MSCs features and capacities, such as in their differentiation potential, whose impact on the therapeutic properties desired for specific diseases requires further investigation.

## Supporting information

S1 Table UCB samples analysis. Initial WBC count, performed before the volume reduction with the AXP automated system (cells x 10^9^ per liter) and the final WBC count (cells x 10^9^ per liter), performed after the volume reduction procedure of the UCB samples, values obtained by using the hematology auto-analyser (Ac T diff2™, Beckman Coulter, Inc.). Total number of viable leucocytes (CD45^+^) per liter, the percentage (%) of viable leucocytes (CD45^+^), the total number of viable CD34^+^ cells per μl, and the % of viable CD34^+^, measured by flow cytometry of the UCB samples used for ^1^H-NMR analysis. The average (Mean), the maximum (Max) and minimum (Min) values, standard deviation (SD) and the standard error of the mean (SEM) of N = 13 are also presented. *Count using the hematology auto-analyzer. #Extrapolated values considering that the collected hUCBP volume comprises of approximately 1/3 of the volume remaining after the removal of the Buffy coat layer, Microbiological analysis were performed after volume reduction and before cryopreservation and tested for microbiological contamination using an automated blood culture system (BacT/ALERT^®^, BioMérieux) at 35°C for 14 days.
(DOCX)

S2 Table Corrected absorbance assessed by PrestoBlue viability assay of hMSCs (UC-MSCs and DPSCs), in the presence of supplemented medium with FBS_II or variable concentrations of hUCBP for up to 9 days. Results presented as Mean ± SEM.
(DOCX)

S3 Table β-Galactosidase activity assay (OD_405nm_) on UC-MSCs and DPSCs at 3, 5 and 7 days. Results Presented as Mean ± SEM.
(DOCX)

S4 Table Annexin V/ PI detection on UC-MSCs and DPSCs after 5 days of culture in hUCBP or FBS supplemented media. Results Presented as Mean ± SEM.
(DOCX)

S5 Table Total RNA extracted from UC-MSCs and DPSCs cultured in hUCBP or FBS supplemented media, readings at 260 and 280 nm. (DOCX)

S6 Table Quantitative PCR of UC-MSCs and DPSCs cultured in hUCBP or FBS supplemented media. Avg ΔCq: average quantification cycle (differential expression of target and housekeeping genes); ΔΔCq: differential expression of sample (4%, 6% and 8% hUCBP) and reference sample (FBS 10%) genes; RQ: relative quantification (fold change compared to the FBS 10% group), in mean fold change ± SEM; nd: not detected; na: not applicable; ↑: up-regulated over 2-fold; ↓: down-regulated under 0.5 fold.
(DOCX)

S7 Table Osteogenic differentiation. Alizarin Red S concentration (μM) after 21 days. Control: Undifferentiated control; Osteo Diff: Osteogenic Differentiation. Results Presented as Mean ± SEM.
(DOCX)

S8 Table Statistical significance in Alizarin Red S concentration (μM) after 21 days. C: Undifferentiated control; D: Osteogenic Differentiation. Significance of the results is indicated according to P values with one, two, three or four of the symbols (*) corresponding to 0.01≤P<0.05; 0.001≤P< 0.01; 0.0001≤P<0.001 and P<0.0001, respectively; ns, not significant.
(DOCX)

S9 Table Adipogenic differentiation. Oil Red O (OD_570nm_) after 14 days. Control: Undifferentiated control; Adipo Diff: Adipogenic Differentiation. Results Presented as Mean ± SEM.
(DOCX)

S10 Table Statistical significance in Oil Red O (OD_570nm_) after 14 days. C: Undifferentiated control; D: Adipogenic Differentiation. Significance of the results is indicated according to P values with one, two, three or four of the symbols (*) corresponding to 0.01≤P<0.05; 0.001≤P< 0.01; 0.0001≤P<0.001 and P<0.0001, respectively; ns, not significant.
(DOCX)

S11 Table Chondrogenic differentiation. Sulfated GAGs production (μg/ml) after 14 days, assessed by Blyscan Glycosaminoglycan Assay (Biocolor, UK). Control: Undifferentiated control; Chondro Diff: Chondrogenic Differentiation. Results Presented as Mean ± SEM.
(DOCX)

S12 Table Statistical significance differences in sulfated GAGs production (μg/ml) after 14 days, assessed by Blyscan Glycosaminoglycan Assay (Biocolor, UK). C: Undifferentiated control; D: Chondrogenic Differentiation. Significance of the results is indicated according to P values with one, two, three or four of the symbols (*) corresponding to 0.01≤P<0.05; 0.001≤P< 0.01; 0.0001≤P<0.001 and P<0.0001, respectively; ns, not significant.
(DOCX)
