# Supplementary material for: Human umbilical cord blood plasma as an alternative to animal sera for mesenchymal stromal cells in vitro expansion – A multicomponent metabolomic analysis
Source: PLoS One. 2018 Oct 10;13(10):e0203936. doi: 10.1371/journal.pone.0203936 (PMC6179201; doi:10.1371/journal.pone.0203936)
Supplement: S1 Table — Initial WBC count, performed before the volume reduction with the AXP automated system (cells x 109 per liter) and the final WBC count (cells x 109 per liter), performed after the volume reduction procedure of the UCB samples, values obtained by using the hematology auto-analyser (Ac T diff2™, Beckman Coulter, Inc.). Total number of viable leucocytes (CD45+) per liter, the percentage (%) of viable leucocytes (CD45+), the total number of viable CD34+ cells per μl, and the % of viable CD34+, measured by flow cytometry of the UCB samples used for 1H-NMR analysis. The average (Mean), the maximum (Max) and minimum (Min) values, standard deviation (SD) and the standard error of the mean (SEM) of N = 13 are also presented. *Count using the hematology auto-analyzer. #Extrapolated values considering that the collected hUCBP volume comprises of approximately 1/3 of the volume remaining after the removal of the Buffy coat layer, Microbiological analysis were performed after volume reduction and before cryopreservation and tested for microbiological contamination using an automated blood culture system (BacT/ALERT®, BioMérieux) at 35°C for 14 days. (DOCX) [file pone.0203936.s001.docx]

| ***hUCBP sample*** | ***Initial WBC***  ***(cells x 10^9^/L)**** | ***Final WBC***  ***(cells x 10^9^/L)**** | ***Total viable CD45^+^ (cells x 10^9^/L)*** | ***Cell viability (CD45^+^)*** | ***Total viable CD34^+^ (cells x 10^9^/L)*** | ***Cell viability (CD34^+^)*** | ***Processed hUCBP volume (mL)*** | ***Collected hUCBP volume (mL)^#^*** | ***Microbiological analysis***  ***(aerobic, anaerobic and fungi)*** |
| --- | --- | --- | --- | --- | --- | --- | --- | --- | --- |
| *hUCBP#1* | 8,15 | 12,45 | 10,00 | 95,39 | 40,00 | 95,68 | 51,06 | 10,15 | negative |
| *hUCBP#2* | 7,00 | 17,30 | 14,30 | 97,99 | 43,33 | 94,08 | 70,10 | 16,47 | negative |
| *hUCBP#3* | 8,50 | 17,80 | 10,80 | 85,19 | 66,34 | 95,18 | 59,24 | 12,81 | negative |
| *hUCBP#4* | 15,70 | 52,60 | 27,50 | 89,32 | 158,49 | 95,05 | 78,92 | 19,34 | negative |
| *hUCBP#5* | 8,00 | 25,80 | 15,80 | 97,87 | 44,93 | 97,26 | 84,09 | 21,13 | *Actinomyces meyeri* |
| *hUCBP#6* | 15,30 | 54,80 | 29,90 | 84,80 | 17,66 | 96,86 | 98,27 | 25,79 | negative |
| *hUCBP#7* | 7,00 | 37,60 | 19,90 | 90,98 | 27,29 | 98,56 | 124,10 | 34,37 | negative |
| *hUCBP#8* | 11,10 | 43,90 | 27,90 | 85,41 | 168,05 | 98,85 | 125,23 | 34,81 | negative |
| *hUCBP#9* | 6,80 | 12,60 | 9,10 | 91,91 | 29,34 | 80,90 | 47,30 | 8,87 | negative |
| *hUCBP#10* | 8,30 | 34,65 | 19,60 | 87,22 | 40,57 | 95,83 | 105,53 | 28,24 | negative |
| *hUCBP#11* | 8,70 | 15,60 | 10,90 | 90,65 | 17,02 | 86,67 | 51,44 | 10,21 | negative |
| *hUCBP#12* | 7,80 | 16,40 | 9,90 | 88,46 | 15,72 | 77,56 | 60,54 | 13,21 | negative |
| *hUCBP#13* | 12,60 | 40,95 | 24,40 | 81,12 | 191,45 | 98,25 | 87,61 | 22,27 | negative |
| ***Mean*** | **9,61** | **29,42** | **17,63** | **89,72** | **66,17** | **93,13** | **80,26** | **19,82** |  |
| ***SD*** | **2,96** | **14,79** | **7,27** | **4,96** | **60,19** | **6,65** | **26,90** | **8,95** |  |
| ***SEM*** | **2,50** | **13,54** | **6,56** | **4,08** | **49,18** | **5,27** | **7,46** | **2,48** |  |
| ***Max*** | **15,70** | **54,80** | **29,09** | **97,99** | **191,45** | **98,85** | **34,81** | **34,81** |  |
| ***Min*** | **6,80** | **12,45** | **9,10** | **81,12** | **15,72** | **77,56** | **8,87** | **8,87** |  |
| ***n*** | **13** | **13** | **13** | **13** | **13** | **13** | **13** | **13** |  |

**S1 Table. UCB Samples Analysis.** Initial WBC count, performed before the volume reduction with the AXP automated system (cells x 10^9^ per liter) and the final WBC count (cells x 10^9^ per liter), performed after the volume reduction procedure of the UCB samples, values obtained by using the hematology auto-analyser (Ac T diff2™, Beckman Coulter, Inc.). Total number of viable leucocytes (CD45^+^) per liter, the percentage (%) of viable leucocytes (CD45^+^), the total number of viable CD34^+^ cells per µl, and the % of viable CD34^+^, measured by flow cytometry of the UCB samples used for ^1^H-NMR analysis. The average (Mean), the maximum (Max) and minimum (Min) values, standard deviation (SD) and the standard error of the mean (SEM) of N=13 are also presented. *Count using the hematology auto-analyzer. #Extrapolated values considering that the collected hUCBP volume comprises of approximately 1/3 of the volume remaining after the removal of the Buffy coat layer, Microbiological analysis were performed after volume reduction and before cryopreservation and tested for microbiological contamination using an automated blood culture system (BacT/ALERT^®^, BioMérieux) at 35ᵒC for 14 days.
