# Supplementary material for: Human umbilical cord blood plasma as an alternative to animal sera for mesenchymal stromal cells in vitro expansion – A multicomponent metabolomic analysis
Source: PLoS One. 2018 Oct 10;13(10):e0203936. doi: 10.1371/journal.pone.0203936 (PMC6179201; doi:10.1371/journal.pone.0203936)
Supplement: S2 Table — Results presented as Mean ± SEM. (DOCX) [file pone.0203936.s002.docx]

| ***PrestoBlue® Cell Viability Assay*** | ***UC-MSCs*** | | | | | | | | | | | | | | |
| --- | --- | --- | --- | --- | --- | --- | --- | --- | --- | --- | --- | --- | --- | --- | --- |
|  | ***FBS 10%*** | | | ***hUCBP 2%*** | | | ***hUCBP 4%*** | | | ***hUCBP 6%*** | | | ***hUCBP 8%*** | | |
| ***24 hours (1 day)*** | 0,0086 | ± | 0,0043 | 0,0052 | ± | 0,0006 | 0,0257 | ± | 0,0038 | 0,0119 | ± | 0,0007 | 0,0144 | ± | 0,0009 |
| ***72 hours (3 days)*** | 0,0134 | ± | 0,0003 | 0,0233 | ± | 0,0029 | 0,0226 | ± | 0,0033 | 0,0038 | ± | 0,0022 | 0,0178 | ± | 0,0020 |
| ***120 hours (5 days)*** | 0,1743 | ± | 0,0033 | 0,1333 | ± | 0,0029 | 0,1773 | ± | 0,0134 | 0,1651 | ± | 0,0135 | 0,1299 | ± | 0,0016 |
| ***168 hours (7 days)*** | 0,5022 | ± | 0,0084 | 0,3807 | ± | 0,0206 | 0,5982 | ± | 0,0190 | 0,5288 | ± | 0,0104 | 0,5097 | ± | 0,0030 |
| ***216 hours (9 days)*** | 0,3637 | ± | 0,0055 | 0,2914 | ± | 0,0084 | 0,3531 | ± | 0,0101 | 0,2942 | ± | 0,0098 | 0,2391 | ± | 0,0007 |
|  |  |  |  |  |  |  |  |  |  |  |  |  |  |  |  |
|  | ***DPSCs*** | | | | | | | | | | | | | | |
|  | ***FBS 10%*** | | | ***hUCBP 2%*** | | | ***hUCBP 4%*** | | | ***hUCBP 6%*** | | | ***hUCBP 8%*** | | |
| ***24 hours (1 day)*** | 0,0173 | ± | 0,0006 | 0,0198 | ± | 0,0008 | 0,0179 | ± | 0,0014 | 0,0167 | ± | 0,0010 | 0,0132 | ± | 0,0004 |
| ***72 hours (3 days)*** | 0,0147 | ± | 0,0028 | 0,0377 | ± | 0,0029 | 0,0341 | ± | 0,0015 | 0,0350 | ± | 0,0020 | 0,0288 | ± | 0,0014 |
| ***120 hours (5 days)*** | 0,1557 | ± | 0,0056 | 0,1489 | ± | 0,0004 | 0,1579 | ± | 0,0009 | 0,1626 | ± | 0,0037 | 0,1406 | ± | 0,0038 |
| ***168 hours (7 days)*** | 0,3487 | ± | 0,0076 | 0,0741 | ± | 0,0048 | 0,2712 | ± | 0,0065 | 0,3273 | ± | 0,0090 | 0,3188 | ± | 0,0048 |
| ***216 hours (9 days)*** | 0,2386 | ± | 0,0021 | 0,0829 | ± | 0,0009 | 0,1323 | ± | 0,0054 | 0,1542 | ± | 0,0065 | 0,1748 | ± | 0,0028 |

**S2 Table.** **Corrected absorbance assessed by PrestoBlue^®^ viability assay of hMSCs** (UC-MSCs and DPSCs), in the presence of supplemented medium with FBS_II or variable concentrations of hUCBP for up to 9 days. Results presented as Mean ± SEM.
