# Supplementary material for: Human umbilical cord blood plasma as an alternative to animal sera for mesenchymal stromal cells in vitro expansion – A multicomponent metabolomic analysis
Source: PLoS One. 2018 Oct 10;13(10):e0203936. doi: 10.1371/journal.pone.0203936 (PMC6179201; doi:10.1371/journal.pone.0203936)
Supplement: S3 Table — Results Presented as Mean ± SEM. (DOCX) [file pone.0203936.s003.docx]

| ***β-Galactosidase (OD_405nm_)*** | ***UC-MSCs*** | | | | | | | | | | | |
| --- | --- | --- | --- | --- | --- | --- | --- | --- | --- | --- | --- | --- |
|  | ***hUCBP 4%*** | | | ***hUCBP 6%*** | | | ***hUCBP 8%*** | | | ***FBS 10%*** | | |
| ***3 days*** | 0,145 | ± | 0,005 | 0,146 | ± | 0,000 | 0,143 | ± | 0,001 | 0,141 | ± | 0,001 |
| ***5 days*** | 0,144 | ± | 0,001 | 0,155 | ± | 0,002 | 0,146 | ± | 0,001 | 0,156 | ± | 0,005 |
| ***7 days*** | 0,138 | ± | 0,004 | 0,149 | ± | 0,002 | 0,149 | ± | 0,003 | 0,132 | ± | 0,005 |
|  | ***DPSCs*** | | | | | | | | | | | |
|  | ***hUCBP 4%*** | | | ***hUCBP 6%*** | | | ***hUCBP 8%*** | | | ***FBS 10%*** | | |
| ***3 days*** | 0,136 | ± | 0,000 | 0,137 | ± | 0,000 | 0,137 | ± | 0,001 | 0,145 | ± | 0,003 |
| ***5 days*** | 0,138 | ± | 0,000 | 0,138 | ± | 0,000 | 0,137 | ± | 0,000 | 0,140 | ± | 0,001 |
| ***7 days*** | 0,138 | ± | 0,000 | 0,143 | ± | 0,000 | 0,142 | ± | 0,001 | 0,144 | ± | 0,002 |

**S3 Table.** **β-Galactosidase activity assay (OD_405nm_) on UC-MSCs and DPSCs** at 3, 5 and 7 days. Results Presented as Mean ± SEM.
