# Supplementary material for: Human umbilical cord blood plasma as an alternative to animal sera for mesenchymal stromal cells in vitro expansion – A multicomponent metabolomic analysis
Source: PLoS One. 2018 Oct 10;13(10):e0203936. doi: 10.1371/journal.pone.0203936 (PMC6179201; doi:10.1371/journal.pone.0203936)
Supplement: S4 Table — Results Presented as Mean ± SEM. (DOCX) [file pone.0203936.s004.docx]

| ***Annexin V/ PI detection (% cells)*** | ***UC-MSCs*** | | | | | | | | | | | |
| --- | --- | --- | --- | --- | --- | --- | --- | --- | --- | --- | --- | --- |
|  | ***hUCBP 4%*** | | | ***hUCBP 6%*** | | | ***hUCBP 8%*** | | | ***FBS 10%*** | | |
| ***Late apoptosis (An-PI+)*** | 0,05 | ± | 0,02 | 0,07 | ± | 0,01 | 0,06 | ± | 0,04 | 0,05 | ± | 0,00 |
| ***Dead cells (An+PI+)*** | 0,14 | ± | 0,06 | 0,11 | ± | 0,05 | 0,59 | ± | 0,23 | 0,10 | ± | 0,04 |
| ***Early apoptosis (An+PI-)*** | 0,29 | ± | 0,03 | 0,40 | ± | 0,04 | 6,42 | ± | 0,53 | 0,47 | ± | 0,07 |
| ***Viable cells (An- PI-)*** | 99,50 | ± | 0,10 | 99,40 | ± | 0,06 | 92,93 | ± | 0,52 | 99,37 | ± | 0,13 |
|  | ***DPSCs*** | | | | | | | | | | | |
|  | ***hUCBP 4%*** | | | ***hUCBP 6%*** | | | ***hUCBP 8%*** | | | ***FBS 10%*** | | |
| ***Late apoptosis (An-PI+)*** | 0,03 | ± | 0,02 | 0,03 | ± | 0,01 | 0,02 | ± | 0,01 | 0,02 | ± | 0,02 |
| ***Dead cells (An+PI+)*** | 0,08 | ± | 0,05 | 0,06 | ± | 0,03 | 0,16 | ± | 0,07 | 0,06 | ± | 0,04 |
| ***Early apoptosis (An+PI-)*** | 0,14 | ± | 0,01 | 0,33 | ± | 0,07 | 0,38 | ± | 0,12 | 0,11 | ± | 0,02 |
| ***Viable cells (An- PI-)*** | 99,73 | ± | 0,07 | 99,57 | ± | 0,03 | 99,43 | ± | 0,09 | 99,80 | ± | 0,06 |

**S4 Table.** **Annexin V/ PI detection on UC-MSCs and DPSCs** after 5 days of culture in hUCBP or FBS supplemented media. Results Presented as Mean ± SEM.
