# Supplementary material for: Human umbilical cord blood plasma as an alternative to animal sera for mesenchymal stromal cells in vitro expansion – A multicomponent metabolomic analysis
Source: PLoS One. 2018 Oct 10;13(10):e0203936. doi: 10.1371/journal.pone.0203936 (PMC6179201; doi:10.1371/journal.pone.0203936)
Supplement: S5 Table — (DOCX) [file pone.0203936.s005.docx]

| ***Total extracted RNA*** | | ***Concentration (ng/μg)*** | ***Absorbance ratio***  ***(260/280 nm)*** |
| --- | --- | --- | --- |
| ***UC-MSCs*** | ***hUCBP 4%*** | 400 | 2,04 |
|  | ***hUCBP 6%*** | 386 | 2,05 |
|  | ***hUCBP 8%*** | 372 | 2,44 |
|  | ***FBS 10%*** | 338 | 2,04 |
| ***DPSCs*** | ***hUCBP 4%*** | 196 | 2,09 |
|  | ***hUCBP 6%*** | 210 | 2,02 |
|  | ***hUCBP 8%*** | 202 | 2,10 |
|  | ***FBS 10%*** | 190 | 2,16 |

**S5 Table**. **Total RNA extracted from UC-MSCs and DPSCs** cultured in hUCBP or FBS supplemented media, readings at 260 and 280 nm.
