# Supplementary material for: Human umbilical cord blood plasma as an alternative to animal sera for mesenchymal stromal cells in vitro expansion – A multicomponent metabolomic analysis
Source: PLoS One. 2018 Oct 10;13(10):e0203936. doi: 10.1371/journal.pone.0203936 (PMC6179201; doi:10.1371/journal.pone.0203936)
Supplement: S6 Table — Avg ΔCq: average quantification cycle (differential expression of target and housekeeping genes); ΔΔCq: differential expression of sample (4%, 6% and 8% hUCBP) and reference sample (FBS 10%) genes; RQ: relative quantification (fold change compared to the FBS 10% group), in mean fold change ± SEM; nd: not detected; na: not applicable; ↑: up-regulated over 2-fold; ↓: down-regulated under 0.5 fold. (DOCX) [file pone.0203936.s006.docx]

| ***UC-MSCs*** | | | | | | | | | | | | | | | | | | | |
| --- | --- | --- | --- | --- | --- | --- | --- | --- | --- | --- | --- | --- | --- | --- | --- | --- | --- | --- | --- |
| ***Target gene*** | ***FBS 10%*** | ***hUCBP 4%*** | | | | | | ***hUCBP 6%*** | | | | | | ***hUCBP 8%*** | | | | | |
|  | ***Avg ΔCq*** | ***Avg ΔCq*** | ***ΔΔCq*** | ***RQ*** | | | | ***Avg ΔCq*** | ***ΔΔCq*** | ***RQ*** | | | | ***Avg ΔCq*** | ***ΔΔCq*** | ***RQ*** | | | |
| ***CD34*** | nd | nd | na | na | | | | nd | na | na | | | | nd | na | na | | | |
| ***CD105*** | 6,8 | 6,0 | -0,7 | 0,6 | ± | 0,1 |  | 5,8 | -0,9 | 0,5 | ± | 0,0 |  | 6,2 | -0,6 | 0,7 | ± | 0,1 |  |
| ***CD117*** | 12,1 | 11,8 | -0,4 | 0,8 | ± | 0,0 |  | 12,4 | 0,2 | 1,2 | ± | 0,0 |  | 12,4 | 0,2 | 1,2 | ± | 0,2 |  |
| ***Sox2*** | nd | nd | na | na | | | | nd | na | na | | | | nd | na | na | | | |
| ***CD166*** | 6,0 | 6,3 | 0,3 | 1,2 | ± | 0,0 |  | 6,0 | 0,0 | 1,0 | ± | 0,0 |  | 6,4 | 0,4 | 1,3 | ± | 0,0 |  |
| ***CD90*** | 4,2 | 2,9 | -1,4 | 0,4 | ± | 0,0 | ↓ | 2,8 | -1,4 | 0,4 | ± | 0,0 | ↓ | 3,0 | -1,3 | 0,4 | ± | 0,0 | ↓ |
| ***CD73*** | 6,8 | 6,8 | 0,0 | 1,0 | ± | 0,1 |  | 6,6 | -0,3 | 0,8 | ± | 0,0 |  | 6,9 | 0,1 | 1,0 | ± | 0,0 |  |
| ***MHC I*** | 5,7 | 3,7 | -2,0 | 0,2 | ± | 0,0 | ↓ | 3,9 | -1,8 | 0,3 | ± | 0,0 | ↓ | 3,9 | -1,8 | 0,3 | ± | 0,0 | ↓ |
| ***MHC II*** | nd | 18,4 | na | na | | | ↑ | 19,3 | na | na | | | ↑ | 15,9 | na | na | | | ↑ |
| ***OCT 4*** | 15,6 | 15,3 | -0,3 | 0,8 | ± | 0,2 |  | 14,0 | -1,6 | 0,3 | ± | 0,1 | ↓ | 14,2 | -1,4 | 0,4 | ± | 0,1 | ↓ |
|  |  |  |  |  |  |  |  |  |  |  |  |  |  |  |  |  |  |  |  |
| ***DPSCs*** | | | | | | | | | | | | | | | | | | | |
| ***Target gene*** | ***FBS 10%*** | ***hUCBP 4%*** | | | | | | ***hUCBP 6%*** | | | | | | ***hUCBP 8%*** | | | | | |
|  | ***Avg ΔCq*** | ***Avg ΔCq*** | ***ΔΔCq*** | ***RQ*** | | | | ***Avg ΔCq*** | ***ΔΔCq*** | ***RQ*** | | | | ***Avg ΔCq*** | ***ΔΔCq*** | ***RQ*** | | | |
| ***CD34*** | nd | nd | na | na | | | | nd | na | na | | |  | nd | na | na | | | |
| ***CD105*** | 7,1 | 6,9 | -0,2 | 0,9 | ± | 0,0 |  | 6,9 | -0,2 | 0,9 | ± | 0,1 |  | 7,7 | 0,6 | 1,5 | ± | 0,0 |  |
| ***CD117*** | 11,3 | 10,2 | -1,1 | 0,5 | ± | 0,0 |  | 10,8 | -0,5 | 0,7 | ± | 0,0 |  | 11,1 | -0,2 | 0,9 | ± | 0,3 |  |
| ***Sox2*** | nd | nd | na | na | | | | nd | na | na | | | | nd | na | na | | | |
| ***CD166*** | 4,6 | 4,5 | -0,1 | 1,0 | ± | 0,0 |  | 4,7 | 0,2 | 1,1 | ± | 0,0 |  | 4,9 | 0,3 | 1,3 | ± | 0,3 |  |
| ***CD90*** | 5,9 | 5,9 | -0,1 | 1,0 | ± | 0,0 |  | 5,8 | -0,1 | 0,9 | ± | 0,1 |  | 6,5 | 0,6 | 1,5 | ± | 0,0 |  |
| ***CD73*** | 4,6 | 5,7 | 1,1 | 2,1 | ± | 0,2 | ↑ | 5,9 | 1,2 | 2,3 | ± | 0,1 | ↑ | 6,6 | 2,0 | 4,0 | ± | 0,2 | ↑ |
| ***MHC I*** | 4,3 | 3,3 | -1,0 | 0,5 | ± | 0,0 |  | 3,2 | -1,1 | 0,5 | ± | 0,0 |  | 3,6 | -0,7 | 0,6 | ± | 0,1 |  |
| ***MHC II*** | 11,2 | 8,9 | -2,3 | 0,2 | ± | 0,0 | ↓ | 9,4 | -1,8 | 0,3 | ± | 0,0 | ↓ | 9,9 | -1,4 | 0,4 | ± | 0,1 | ↓ |
| ***OCT 4*** | 14,2 | 15,8 | 1,5 | 2,9 | ± | 0,4 | ↑ | 16,3 | 2,1 | 4,2 | ± | 0,0 | ↑ | 15,5 | 1,3 | 2,5 | ± | 1,5 | ↑ |

**S6 Table.** **Quantitative PCR of UC-MSCs and DPSCs** cultured in hUCBP or FBS supplemented media. Avg ΔCq: average quantification cycle (differential expression of target and housekeeping genes); ΔΔCq: differential expression of sample (4%, 6% and 8% hUCBP) and reference sample (FBS 10%) genes; RQ: relative quantification (fold change compared to the FBS 10% group), in mean fold change ± SEM; nd: not detected; na: not applicable; ↑: up-regulated over 2-fold; ↓: down-regulated under 0.5 fold.
