# Supplementary material for: Human umbilical cord blood plasma as an alternative to animal sera for mesenchymal stromal cells in vitro expansion – A multicomponent metabolomic analysis
Source: PLoS One. 2018 Oct 10;13(10):e0203936. doi: 10.1371/journal.pone.0203936 (PMC6179201; doi:10.1371/journal.pone.0203936)
Supplement: S7 Table — Alizarin Red S concentration (μM) after 21 days. Control: Undifferentiated control; Osteo Diff: Osteogenic Differentiation. Results Presented as Mean ± SEM. (DOCX) [file pone.0203936.s007.docx]

| ***Alizarin Red S***  ***(μM)*** | ***UC-MSCs*** | | | | | | | | | | | |
| --- | --- | --- | --- | --- | --- | --- | --- | --- | --- | --- | --- | --- |
|  | ***hUCBP 4%*** | | | ***hUCBP 6%*** | | | ***hUCBP 8%*** | | | ***FBS 10%*** | | |
| ***Osteo Diff*** | 68,45 | ± | 1,20 | 74,48 | ± | 0,32 | 72,14 | ± | 0,27 | 1449,57 | ± | 0,00 |
| ***Control*** | 81,85 | ± | 0,00 | 87,21 | ± | 0,00 | 78,72 | ± | 0,45 | 89,44 | ± | 0,45 |
|  |  |  |  |  |  |  |  |  |  |  |  |  |
|  | ***DPSCs*** | | | | | | | | | | | |
|  | ***hUCBP 4%*** | | | ***hUCBP 6%*** | | | ***hUCBP 8%*** | | | ***FBS 10%*** | | |
| ***Osteo Diff*** | 68,79 | ± | 0,82 | 71,13 | ± | 0,00 | 73,81 | ± | 0,00 | 2299,31 | ± | 7,35 |
| ***Control*** | 61,31 | ± | 0,45 | 62,65 | ± | 0,45 | 60,86 | ± | 0,45 | 68,45 | ± | 0,00 |

**S7 Table. Osteogenic differentiation.** Alizarin Red S concentration (μM) after 21 days. Control: Undifferentiated control; Osteo Diff: Osteogenic Differentiation. Results Presented as Mean ± SEM.
