# Supplementary material for: Human umbilical cord blood plasma as an alternative to animal sera for mesenchymal stromal cells in vitro expansion – A multicomponent metabolomic analysis
Source: PLoS One. 2018 Oct 10;13(10):e0203936. doi: 10.1371/journal.pone.0203936 (PMC6179201; doi:10.1371/journal.pone.0203936)
Supplement: S9 Table — Oil Red O (OD570nm) after 14 days. Control: Undifferentiated control; Adipo Diff: Adipogenic Differentiation. Results Presented as Mean ± SEM. (DOCX) [file pone.0203936.s009.docx]

| ***Oil Rel O***  ***(OD _570 nm_)*** | ***UC-MSCs*** | | | | | | | | | | | |
| --- | --- | --- | --- | --- | --- | --- | --- | --- | --- | --- | --- | --- |
|  | ***hUCBP 4%*** | | | ***hUCBP 6%*** | | | ***hUCBP 8%*** | | | ***FBS 10%*** | | |
| ***Adipo Diff*** | 0,158 | ± | 0,007 | 0,149 | ± | 0,001 | 0,151 | ± | 0,004 | 0,164 | ± | 0,001 |
| ***Control*** | 0,121 | ± | 0,007 | 0,107 | ± | 0,003 | 0,113 | ± | 0,001 | 0,077 | ± | 0,001 |
|  |  |  |  |  |  |  |  |  |  |  |  |  |
|  | ***DPSCs*** | | | | | | | | | | | |
|  | ***hUCBP 4%*** | | | ***hUCBP 6%*** | | | ***hUCBP 8%*** | | | ***FBS 10%*** | | |
| ***Adipo Diff*** | 0,158 | ± | 0,007 | 0,149 | ± | 0,001 | 0,151 | ± | 0,004 | 0,164 | ± | 0,001 |
| ***Control*** | 0,121 | ± | 0,007 | 0,107 | ± | 0,003 | 0,113 | ± | 0,001 | 0,077 | ± | 0,001 |

**S9 Table. Adipogenic differentiation.** Oil Red O (OD_570nm_) after 14 days. Control: Undifferentiated control; Adipo Diff: Adipogenic Differentiation. Results Presented as Mean ± SEM.
