# Supplementary material for: Human umbilical cord blood plasma as an alternative to animal sera for mesenchymal stromal cells in vitro expansion – A multicomponent metabolomic analysis
Source: PLoS One. 2018 Oct 10;13(10):e0203936. doi: 10.1371/journal.pone.0203936 (PMC6179201; doi:10.1371/journal.pone.0203936)
Supplement: S11 Table — Sulfated GAGs production (μg/ml) after 14 days, assessed by Blyscan Glycosaminoglycan Assay (Biocolor, UK). Control: Undifferentiated control; Chondro Diff: Chondrogenic Differentiation. Results Presented as Mean ± SEM. (DOCX) [file pone.0203936.s011.docx]

| ***GAGs content***  ***(µg/mL)*** | ***UC-MSCs*** | | | | | | | | | | | |
| --- | --- | --- | --- | --- | --- | --- | --- | --- | --- | --- | --- | --- |
|  | ***hUCBP 4%*** | | | ***hUCBP 6%*** | | | ***hUCBP 8%*** | | | ***FBS 10%*** | | |
| ***Chondro Diff*** | 7,92 | ± | 0,86 | 6,74 | ± | 0,79 | 7,91 | ± | 0,87 | 6,91 | ± | 0,39 |
| ***Control*** | 5,32 | ± | 0,58 | 4,84 | ± | 0,43 | 4,97 | ± | 0,16 | 2,15 | ± | 0,62 |
|  | | | | | | | | | | | | |
|  | ***DPSCs*** | | | | | | | | | | | |
|  | ***hUCBP 4%*** | | | ***hUCBP 6%*** | | | ***hUCBP 8%*** | | | ***FBS 10%*** | | |
| ***Chondro Diff*** | 7,23 | ± | 0,36 | 7,56 | ± | 0,23 | 6,74 | ± | 0,16 | 8,29 | ± | 0,58 |
| ***Control*** | 2,80 | ± | 0,17 | 3,31 | ± | 0,06 | 3,07 | ± | 0,77 | 3,03 | ± | 0,02 |

**S11 Table. Chondrogenic differentiation.** Sulfated GAGs production (μg/ml) after 14 days, assessed by Blyscan™ Glycosaminoglycan Assay (Biocolor, UK). Control: Undifferentiated control; Chondro Diff: Chondrogenic Differentiation. Results Presented as Mean ± SEM.
