# Supplementary material for: Human umbilical cord blood plasma as an alternative to animal sera for mesenchymal stromal cells in vitro expansion – A multicomponent metabolomic analysis
Source: PLoS One. 2018 Oct 10;13(10):e0203936. doi: 10.1371/journal.pone.0203936 (PMC6179201; doi:10.1371/journal.pone.0203936)
Supplement: S12 Table — C: Undifferentiated control; D: Chondrogenic Differentiation. Significance of the results is indicated according to P values with one, two, three or four of the symbols (*) corresponding to 0.01≤P<0.05; 0.001≤P< 0.01; 0.0001≤P<0.001 and P<0.0001, respectively; ns, not significant. (DOCX) [file pone.0203936.s012.docx]

| ***GAGs Assay***  ***Statistical significance*** | |  | ***UC-MSCs*** | | | | | | | | ***DPSCs*** | | | | | | | |
| --- | --- | --- | --- | --- | --- | --- | --- | --- | --- | --- | --- | --- | --- | --- | --- | --- | --- | --- |
|  |  |  | ***hUCBP 4%*** | | ***hUCBP 6%*** | | ***hUCBP 8%*** | | ***FBS 10%*** | | ***hUCBP 4%*** | | ***hUCBP 6%*** | | ***hUCBP 8%*** | | ***FBS 10%*** | |
|  |  |  | D | C | D | C | D | C | D | C | D | C | D | C | D | C | D | C |
| ***hUC-MSCs*** | ***hUCBP 4%*** | D |  | ns | ns | * | ns | * | ns | **** | ns | *** | ns | ** | ns | ** | ns | ** |
|  |  | C |  |  | ns | ns | ns | ns | ns | * | ns | ns | ns | ns | ns | ns | ns | ns |
|  | ***hUCBP 6%*** | D |  |  |  | ns | ns | ns | ns | ns | ns | * | ns | ns | ns | * | ns | * |
|  |  | C |  |  |  |  | * | ns | ns | ns | ns | ns | ns | ns | ns | ns | ns | ns |
|  | ***hUCBP 8%*** | D |  |  |  |  |  | ns | ns | ns | ns | *** | ns | ** | ns | ** | ns | ** |
|  |  | C |  |  |  |  |  |  | ns | ns | ns | ns | ns | ns | ns | ns | ns | ns |
|  | ***FBS 10%*** | D |  |  |  |  |  |  |  | ** | ns | * | ns | ns | ns | * | ns | * |
|  |  | C |  |  |  |  |  |  |  |  | ** | ns | *** | ns | ** | ns | **** | ns |
| ***hDPSCs*** | ***hUCBP 4%*** | D |  |  |  |  |  |  |  |  |  | * | ns | ns | ns | ns | ns | * |
|  |  | C |  |  |  |  |  |  |  |  |  |  | * | ns | ns | ns | ** | ns |
|  | ***hUCBP 6%*** | D |  |  |  |  |  |  |  |  |  |  |  | * | ns | * | ns | * |
|  |  | C |  |  |  |  |  |  |  |  |  |  |  |  | ns | ns | ** | ns |
|  | ***hUCBP 8%*** | D |  |  |  |  |  |  |  |  |  |  |  |  |  | ns | ns | ns |
|  |  | C |  |  |  |  |  |  |  |  |  |  |  |  |  |  | ** | ns |
|  | ***FBS 10%*** | D |  |  |  |  |  |  |  |  |  |  |  |  |  |  |  | ** |
|  |  | C |  |  |  |  |  |  |  |  |  |  |  |  |  |  |  |  |

**S12 Table.** **Statistical Significance Differences in Sulfated GAGs production (μg/ml)** after 14 days, assessed by Blyscan™ Glycosaminoglycan Assay (Biocolor, UK). C: Undifferentiated control; D: Chondrogenic Differentiation. Significance of the results is indicated according to P values with one, two, three or four of the symbols (*) corresponding to 0.01≤P<0.05; 0.001≤P< 0.01; 0.0001≤P<0.001 and P<0.0001, respectively; ns, not significant.
